# Vitamin D Supplementation, Total Testosterone, and Androgen Bioavailability Markers in Adult Men: A Systematic Review and Meta-Analysis of Randomized Controlled Trials

**DOI:** 10.3390/nu18132090

**Published:** 2026-06-26

**Authors:** Loreto Paez-Allendes, Juan José Valenzuela-Fuenzalida, María P. Moya, Gustavo Oyanedel, Gloria Cifuentes-Suazo, Julio Figueroa-Puig, Mathias Orellana-Donoso, Eduardo Mateluna-Valls, Juan Jose Cabezas-Salgado, Juan Sanchis-Gimeno, Alejandro Bruna-Mejias

**Affiliations:** 1 Departamento de Ciencias y Geografía, Facultad de Ciencias Naturales y Exactas, Universidad de Playa Ancha, Valparaíso 2360072, Chile; loreto.paez@upla.cl; 2 Escuela de Medicina, Facultad de Medicina, Universidad Andrés Bello, Viña del Mar 2520000, Chile; juan.kine.2015@gmail.com; 3 Departamento de Ciencias Química y Biológicas, Facultad de Ciencias de la Salud, Universidad Bernardo O’Higgins, Santiago 8370993, Chile; 4 Facultad de Ciencias de la Salud, Universidad Autónoma de Chile, Santiago 8910060, Chile; maria.moya@uautonoma.cl; 5 Faculty of Health and Social Sciences, University of the Americas, Santiago 8370040, Chile; g.oyanedelamaro@gmail.com; 6 Facultad de Medicina, Carrera de Odontología, Universidad Católica de la Santísima Concepción, Av. Alonso de Ribera 2850, Concepción 4090541, Chile; gbcifuentess@gmail.com; 7 Escuela de Kinesiología, Facultad de Medicina, Universidad Mayor, Santiago 7500000, Chile; jifpuig@gmail.com; 8 Escuela de Medicina, Universidad Finis Terrae, Santiago 7501015, Chile; mathor94@gmail.com; 9 Facultad de Medicina y Ciencia, Universidad San Sebastián, Lota 2465, Santiago 7510157, Chile; 10 Académico Carrera de Kinesiología, Departamento de Salud, Comunidad y Gestión, Facultad de Ciencia de la Salud, Universidad de Playa Ancha, Valparaíso 2340000, Chile; eduardo.mateluna@upla.cl; 11 Facultad de Medicina, Universidad Católica del Maule, Talca 3460000, Chile; jjcabezas@gmail.com; 12 GIAVAL Research Group, Department of Anatomy and Human Embryology, Faculty of Medicine, University of Valencia, 46001 Valencia, Spain; juan.sanchis@uv.es; 13 Programa de Doctorado en Investigación Aplicada a las Ciencias Sanitarias, Escuela de Doctorado, Universidad de Las Palmas de Gran Canaria, 35001 Las Palmas de Gran Canaria, Spain

**Keywords:** vitamin D, cholecalciferol, testosterone, free testosterone, sex hormone-binding globulin, free androgen index, androgen bioavailability, male hypogonadism, systematic review, meta-analysis

## Abstract

Background: Vitamin D has traditionally been recognized for its role in calcium homeostasis and skeletal health, but vitamin D receptor expression and vitamin D-metabolizing enzymes have also been identified in extra-skeletal tissues, including components of the male reproductive tract. Observational evidence has suggested associations between vitamin D status and androgen-related markers; however, whether vitamin D supplementation has a measurable effect on androgen bioavailability remains uncertain. Objective: This systematic review and meta-analysis evaluated the effects of vitamin D supplementation on total testosterone (TT) and androgen bioavailability markers in adult men, including sex hormone-binding globulin (SHBG), free androgen index (FAI), calculated free testosterone (calculated FT), and bioactive testosterone (BAT) where methodologically compatible. Methods: The review was registered in PROSPERO (CRD420261365005) and conducted according to PRISMA 2020 and Cochrane methodological guidance. Searches were conducted from database inception to April 2026 in PubMed, Web of Science, Scopus, ClinicalTrials.gov, and the WHO ICTRP. Embase was initially planned but was not searched because institutional access was unavailable; this amendment was made before screening, extraction, risk-of-bias assessment, and synthesis. Records were deduplicated in Zotero, screened in a structured matrix, and converted from report-level records into independent comparison-level datasets where appropriate. Meta-analyses used random-effects REML models with Hartung–Knapp adjustment. Results: The official search set comprised 2854 records, of which 703 duplicates were removed, leaving 2151 records for title and abstract screening. The full-text screening file was reconciled to 162 PRISMA-countable reports/records: 135 reports were assessed, 27 reports could not be assessed because the full text was unavailable or had not been obtained for review, and 27 reports/studies were retained for qualitative synthesis. Eighteen reports were considered candidate sources for quantitative synthesis and were operationalized into 21 comparison-level records. The primary TT model included 11 comparisons and showed no clear effect of vitamin D supplementation on final TT (MD 0.47 nmol/L, 95% CI −0.50 to 1.44; *I*^2^ = 24.1%). No clear effects were observed for SHBG (MD 0.27 nmol/L, 95% CI −2.14 to 2.68), FAI (MD −0.37, 95% CI −4.28 to 3.55), calculated FT sensitivity evidence (MD −0.0096 nmol/L, 95% CI −0.0525 to 0.0332), or BAT exploratory evidence (MD −0.47 nmol/L, 95% CI −1.77 to 0.83). GRADE certainty was low for TT, SHBG, and FAI, and very low for calculated FT and BAT. Conclusions: Current randomized evidence does not demonstrate a statistically clear or reproducible effect of vitamin D supplementation on total testosterone or androgen bioavailability markers in adult men. GRADE certainty was low for total testosterone, SHBG, and FAI, and very low for calculated free testosterone and bioactive testosterone. Because directly measured and calculated free testosterone are not analytically equivalent, free testosterone was not pooled as a primary outcome; method-compatible calculated FT was handled as sensitivity evidence and BAT as exploratory evidence.

## 1. Introduction

Vitamin D is a secosteroid hormone obtained through cutaneous ultra-violet-B exposure, diet, and supplementation. Although its established role is calcium phosphate homeostasis and skeletal health, vitamin D signaling is also relevant to several extra-skeletal systems. Recent consensus and guideline documents emphasize that serum 25-hydroxyvitamin D [25(OH)D] is the usual marker of vitamin D status, but that supplementation effects depend strongly on baseline status, assay standardization, dose, formulation, adherence, and population context [1,2,3,4,5,6]. This context is important when evaluating endocrine outcomes because null or inconsistent trial findings may reflect biological absence of effect, inadequate targeting of deficient populations, heterogeneous intervention designs, or outcome measurement variability.

Male androgen physiology is also methodologically complex. Total testosterone (TT) is the most commonly reported marker, but androgen action is influenced by sex hormone-binding globulin (SHBG), albumin binding, and the free or weakly albumin-bound fractions of circulating testosterone [7,8,9,10]. Consequently, TT, SHBG, free androgen index (FAI), calculated free testosterone (calculated FT), directly measured FT, and bioactive or bioavailable testosterone do not represent interchangeable endpoints. Table 1 provides a concise conceptual summary of how these markers were interpreted in the present review.

A biological link between vitamin D and male reproductive endocrinology is plausible, but it should not be overstated. Vitamin D receptor expression and vitamin D-metabolizing enzymes have been reported in human male reproductive tissues, including testicular cells and spermatozoa [11,12]. Experimental and translational evidence also indicates that Leydig-cell steroidogenesis is sensitive to aging, metabolic dysfunction, inflammatory stress, and changes in the testicular niche [13,14]. These mechanisms provide a rationale for testing vitamin D-related androgen hypotheses, but they do not establish that supplementation will reliably increase circulating androgen concentrations in adult men.

Observational studies have reported associations between serum 25(OH)D, hypogonadism, TT, SHBG, and FAI in men [15,16]. However, observational evidence is vulnerable to confounding by adiposity, insulin resistance, physical activity, sunlight exposure, comorbidity burden, and general health status. Randomized controlled trials are therefore essential to distinguish correlation from treatment effect. Existing trials have yielded mixed findings: some reported increases in TT, calculated FT, BAT, or FAI after supplementation, whereas others showed no meaningful change in TT, SHBG, or FT-related outcomes [17,18,19,20,21,22,23,24,25,26,27,28,29,30,31,32,33].

Previous syntheses have attempted to integrate this evidence, but important limitations remain. A recent meta-analytic review evaluated vitamin D and androgen/anabolic outcomes in adult males [34]. However, for clinical and methodological interpretation, it remains necessary to separate analytically non-equivalent androgen constructs, avoid unit-of-analysis errors in factorial or multi-population trials, distinguish primary, sensitivity, and exploratory evidence, and assess certainty using an explicit framework. These issues are particularly relevant because several androgen outcomes were secondary biomarkers, and because 25(OH)D and testosterone assays varied across trials [3,10,35,36].

Accordingly, this systematic review and meta-analysis evaluated the effects of vitamin D supplementation on TT and androgen bioavailability markers in adult men, including SHBG, FAI, calculated FT, and BAT where methodologically compatible. TT was treated as the most reproducible quantitative anchor because it was the most consistently reported androgen outcome, whereas SHBG, FAI, calculated FT, and BAT were interpreted within the androgen bioavailability framework.

## 2. Materials and Methods

### 2.1. Protocol and Registration

This systematic review and meta-analysis was registered in the International Prospective Register of Systematic Reviews (PROSPERO) in 2026 under the registration number CRD420261365005. The protocol is publicly available at https://www.crd.york.ac.uk/PROSPERO/view/CRD420261365005 (accessed on 5 April 2026). The review was conducted and reported in accordance with the PRISMA 2020 statement [37] and with contemporary methodological guidance for reviews of interventions, including the Cochrane Handbook where applicable [38].

An amendment to the original search plan was made before title and abstract screening, data extraction, risk-of-bias assessment, and synthesis. Embase had initially been planned as a bibliographic source; however, it could not be searched because institutional access was unavailable. This deviation was documented prospectively as a protocol amendment before eligibility or synthesis decisions were made. We acknowledge that absence of Embase may have reduced search sensitivity, particularly for intervention studies variably indexed across biomedical databases. To mitigate this limitation, the final strategy combined three major bibliographic databases (PubMed, Web of Science, and Scopus), two trial registries (ClinicalTrials.gov and WHO ICTRP), broad and targeted search strings, deduplication in Zotero 9.0.4, full-text audit trails, and citation checking of relevant reviews and included reports.

### 2.2. Eligibility Criteria

Eligible studies include intervention studies evaluating vitamin D supplementation in adult men and reporting at least one androgen-related biochemical outcome. Adult men are defined as participants aged 18 years or older. Studies including mixed-sex populations are eligible only when male-specific data are reported separately or can be obtained from the publication.

The intervention of interest is supplementation with vitamin D or vitamin D-related preparations, including cholecalciferol, ergocalciferol, or other vitamin D formulations where relevant. Studies may include any dose, duration, route, or frequency of supplementation. Comparators may include placebo, no supplementation, usual care, or another non-vitamin D control condition.

The outcomes of interest were total testosterone (TT) and androgen bioavailability markers, including sex hormone-binding globulin (SHBG), free androgen index (FAI), free testosterone (FT), and bioactive testosterone (BAT; also referred to as bioavailable testosterone in some reports) where reported. FT was classified according to the method of ascertainment, distinguishing directly measured FT from calculated FT using recognized equations such as Vermeulen. TT was treated as a secondary biochemical anchor because it was the most consistently reported androgen outcome across candidate trials and be-cause it aligns the quantitative synthesis with the main forest plot hierarchy. The interpretative hierarchy therefore remained centered on androgen bioavailability markers, with TT presented first as the most reproducible quantitative anchor.

Studies are excluded if they do not evaluate vitamin D supplementation, do not report an eligible androgen-related outcome, do not include adult men or do not report male-specific data, are conducted exclusively in animals or in vitro models, or correspond to reviews, editorials, letters, conference abstracts without sufficient data, protocols without results, or other non-original research designs. Reviews and secondary publications may be retained for citation tracking but will not contribute to quantitative synthesis.

### 2.3. Information Sources and Search Strategy

The validated search set was derived from PubMed, Web of Science, Scopus, Clin-icalTrials.gov, and WHO ICTRP. Searches were conducted from database inception to April 2026. The search strategy combined terms related to vitamin D exposure or supplementation with terms related to testosterone, free testosterone, SHBG, sex hormone-binding globulin, free androgen index, and androgen bioavailability. Database-specific syntax was adapted according to each platform.

The official PRISMA identification counts validated for this review were: PubMed, 1465 records; Web of Science, 339 records; Scopus, 1005 records; ClinicalTrials.gov, 40 records; and WHO ICTRP, 5 records. Overall, 2854 records were identified. After deduplication, 703 duplicates were removed, leaving 2151 unique records for title and abstract screening. The complete search strategies for each database are provided in Supplementary File S1.

### 2.4. Selection Process and PRISMA Reconciliation

All retrieved records were exported to Zotero for reference management and deduplication. Title and abstract screening was performed independently by A.B.-M., J.J.V.-F., and G.O. Full-text eligibility was subsequently assessed using a standardized decision matrix, with disagreements resolved through discussion and senior methodological adjudication when required. Data extraction was performed by J.J.C.-S., L.P.-A., and E.M.-V. using predefined extraction templates. Risk of bias was assessed independently by J.F.-P. and J.J.C.-S. using the RoB 2 tool at the outcome level [39], with disagreements resolved by consensus. After title/abstract screening and full-text retrieval work, the full-text screening matrix was reconciled at the report level. The final PRISMA-countable full-text universe was set at 162 reports/records after excluding two operational duplicates/overlapping records from the count while retaining their traceability in the audit file.

### 2.5. Study-Level and Comparison-Level Handling

To avoid unit-of-analysis errors, reports were converted into unique studies and, where appropriate, independent analytic comparisons before quantitative extraction. Multi-population trials were split only when the publication reported separate male-specific data by a clinically distinct population. Factorial trials were handled using a single prespecified vitamin D main-effect contrast to avoid double counting. Duplicate or overlapping reports were retained for traceability but not counted twice in PRISMA or quantitative synthesis. These decisions were synthesis-structuring decisions rather than assumptions of biological equivalence; therefore, they were documented in the Supplementary Materials and are interpreted together with sensitivity and exploratory analyses.

For Saha et al. [20], the preferred primary approach was a collapsed factorial vitamin D main-effect contrast, pooling cholecalciferol-containing arms and comparing them with non-cholecalciferol arms. For Ulrich et al. [21], the healthy-subject and hemodialysis cohorts were treated as two clinically distinct comparisons. For Heijboer et al. [19], the three source trials were kept separate but were not directly meta-analyzable for final-value TT because testosterone change was reported as median and IQR. For Jorde et al. [32], data were suitable for change-score contextualization but not the final-value primary TT model because final SDs were not reported. Studies with ambiguous units, internally inconsistent dispersion, or non-comparable outcome scales were retained in the audit trail but excluded from primary pooling to protect scale validity.

### 2.6. Data Extraction and Quantitative Decision Rules

Data extraction was performed using a standardized extraction form. Extracted in-formation includes study identification, country, design, population characteristics, sample size, baseline vitamin D status, baseline androgen status, clinical condition where applicable, vitamin D formulation, dose, route, frequency, intervention duration, comparator, follow-up time points, and outcome measurement methods.

For hormonal outcomes, extracted variables include free testosterone, SHBG, free androgen index, bioavailable testosterone, total testosterone, and other androgen-associated biochemical outcomes when reported. Total testosterone was extracted first because it is the most consistently available androgen outcome and allows early testing of the extraction pipeline before extracting SHBG and bioavailability markers.

Primary total testosterone extraction used final post-intervention values when mean and SD were available, or when SD could be defensibly derived from internally consistent 95% confidence intervals and sample size. Values reported as ng/mL were converted to nmol/L using 1 ng/mL = 3.467 nmol/L. Values reported as median/IQR, figure-only estimates, *p* values only, or narrative statements without extractable dispersion were not used in the primary final-value model. Such studies were retained for qualitative synthesis or considered for prespecified sensitivity analyses only when appropriate.

When final total testosterone was reported as mean with 95% confidence intervals, standard deviations were derived only when the confidence interval was internally consistent with the reported mean and sample size. Studies with internally inconsistent dispersion data were excluded from the primary quantitative synthesis and retained in the qualitative synthesis. When testosterone units were ambiguous or physiologically implausible, the study was excluded from the primary analysis unless unit verification was available; sensitivity analyses were considered only when a biologically plausible unit correction could be transparently justified.

For SHBG, final post-intervention values were synthesized as mean differences in nmol/L. The primary conservative SHBG analysis included studies reporting means and standard deviations directly or after straightforward conversion from standard errors or confidence intervals. Studies reporting medians and interquartile ranges were retained for an all-study sensitivity analysis after conversion to approximate means and standard deviations. Factorial arms were collapsed when needed to estimate a single vitamin D versus no vitamin D contrast and avoid double counting.

For FAI, final post-intervention values were synthesized as mean differences only when scale compatibility was defensible. Because FAI reporting varied across trials, each comparison was classified using a scale-status framework. The primary conservative FAI analysis included studies reporting FAI in a comparable ×100/index scale with mean and SD data directly available or derivable through prespecified rules. Studies requiring conversion from median and interquartile range were retained for sensitivity analysis. Studies with probable simple-ratio reporting or internally inconsistent FAI scaling were excluded from the primary quantitative model to avoid mixing non-equivalent outcome scales.

For bioactive testosterone (BAT), final post-intervention values were synthesized only when compatible derived BAT measures were reported with sufficient dispersion. Because few trials reported BAT and the available evidence was limited to calculated indices, BAT was handled as an exploratory outcome rather than as a primary inferential endpoint. Throughout the manuscript, BAT is used as the preferred term for this outcome; when contributing reports used the term bioavailable testosterone, it was considered only if the construct and units were compatible with BAT.

For free testosterone, studies were first classified by the ascertainment method. Directly measured FT and calculated FT were not pooled together because they reflect analytically distinct constructs and may differ in assay performance, calibration, and biological interpretation. A quantitative synthesis was considered only for method-compatible calculated FT studies, whereas directly measured FT was retained for structured narrative interpretation when no compatible pool was available.

### 2.7. Risk-of-Bias Assessment

Risk of bias in randomized controlled trials was assessed using the revised Cochrane risk-of-bias tool for randomized trials (RoB 2) at the outcome level [39]. Judgments were made specifically for final total testosterone, rather than globally for the article, across five domains: bias arising from the randomization process, bias due to deviations from intended interventions, bias due to missing outcome data, bias in measurement of the outcome, and bias in selection of the reported result.

Across the 11 independent comparisons included in the primary total testosterone analysis, one comparison was judged as low risk of bias, eight as having some concerns, and two as high risk of bias. The most frequent sources of concern were incomplete or per-protocol outcome data, testosterone being assessed as a secondary or exploratory biomarker, subgroup-based male analyses, and potential cointerventions such as structured weight loss, athletic training, calcium co-supplementation, or prior sunlight exposure. The two high-risk comparisons were driven by substantial missing outcome data in a secondary analysis of advanced heart failure patients and by non-prespecified testosterone analysis in a male subgroup of a broader weight-loss trial.

Studies with extractable testosterone data were retained in the primary synthesis even when judged as high risk of bias, but prespecified sensitivity analyses were per-formed excluding high-risk studies. Data validity problems, such as internally inconsistent confidence intervals or ambiguous testosterone units, were treated as extractability issues rather than RoB 2 judgments.

### 2.8. Effect Measures and Data Synthesis

Continuous outcomes were summarized using mean differences when outcomes were reported on the same scale. The primary total testosterone final-value model used nmol/L as the common unit. Testosterone values reported as ng/mL were converted to nmol/L using 1 ng/mL = 3.467 nmol/L. Standard deviations were derived from standard errors or internally consistent 95% confidence intervals only when the required inputs were available and coherent.

For the total testosterone final-value analysis, effect estimates were calculated as mean differences between the vitamin D and control groups. Positive values indicate higher final total testosterone concentrations in the vitamin D group. The primary model used a random-effects framework with restricted maximum likelihood estimation of between-study variance and Hartung–Knapp adjustment for confidence intervals. Heterogeneity was assessed using tau^2^, *I*^2^, and prediction intervals.

Sensitivity analyses were prespecified to evaluate robustness after excluding high-risk-of-bias comparisons, clinically extreme populations, athlete/military/active sport populations, and studies with strong cointerventions. These analyses were interpreted as robustness and context analyses, not as confirmatory subgroup evidence. Factorial or multi-arm trials were handled by combining relevant arms to avoid double counting; multi-population reports were split only when clinically distinct male-specific datasets were reported.

The SHBG primary analysis was conducted using a random-effects REML model with Hartung–Knapp adjustment. Heterogeneity was evaluated using tau^2^, *I*^2^, the Q test, and prediction intervals. A prespecified sensitivity analysis incorporated all extractable SHBG studies, including those converted from medians and interquartile ranges.

The FAI primary conservative analysis was also conducted using a random-effects REML model with Hartung–Knapp adjustment. Heterogeneity was evaluated using tau^2^, *I*^2^, the Q test, and prediction intervals. A prespecified sensitivity analysis incorporated one additional comparison converted from median and interquartile range when scale compatibility was otherwise defensible.

The calculated FT sensitivity analysis and the exploratory BAT analysis were con-ducted using random-effects REML models with Hartung–Knapp adjustment, consistent with the other continuous outcomes. These models were interpreted as secondary or exploratory because the underlying evidence base was limited and method compatibility constrained the eligible comparisons.

All meta-analyses were conducted in RStudio (version 2026.04.0+526) using R and the metafor package. Random-effects models were fitted using restricted maximum likelihood estimation, and confidence intervals were calculated using the Hartung–Knapp adjustment. Forest plots and supplementary figures were generated from the final R-ready datasets.

Small-study effects and publication bias were considered qualitatively. Formal funnel-plot asymmetry tests were not performed because the number of studies contributing to each outcome-specific meta-analysis was below the conventional threshold required for reliable interpretation, and because several outcomes included clinically and methodologically heterogeneous comparisons. This decision was made to avoid overinterpreting underpowered asymmetry assessments.

### 2.9. Certainty of Evidence

The certainty of evidence for each outcome was assessed using the GRADE approach [40]. Randomized evidence was initially rated as high certainty and was downgraded according to concerns regarding risk of bias, inconsistency, indirectness, imprecision, and publication bias. Certainty was assessed separately for total testosterone, SHBG, free androgen index, calculated free testosterone, and bioactive testosterone. Because free testosterone was reported using non-equivalent ascertainment methods across trials, directly measured free testosterone and calculated free testosterone were not pooled together. The GRADE assessment for free testosterone was therefore applied only to the method-restricted sensitivity analysis of calculated free testosterone. Bioactive testosterone was assessed as exploratory because compatible data were available from only two studies.

## 3. Results

### 3.1. Study Selection and Full-Text Audit

The results are presented according to the prespecified outcome hierarchy. TT is reported first because it was the most consistently extractable androgen outcome and provides the main quantitative anchor. SHBG, FAI, calculated FT, and BAT are then presented as androgen bioavailability markers, with calculated FT restricted to method-compatible sensitivity evidence and BAT interpreted as exploratory evidence.

**Figure 1:**
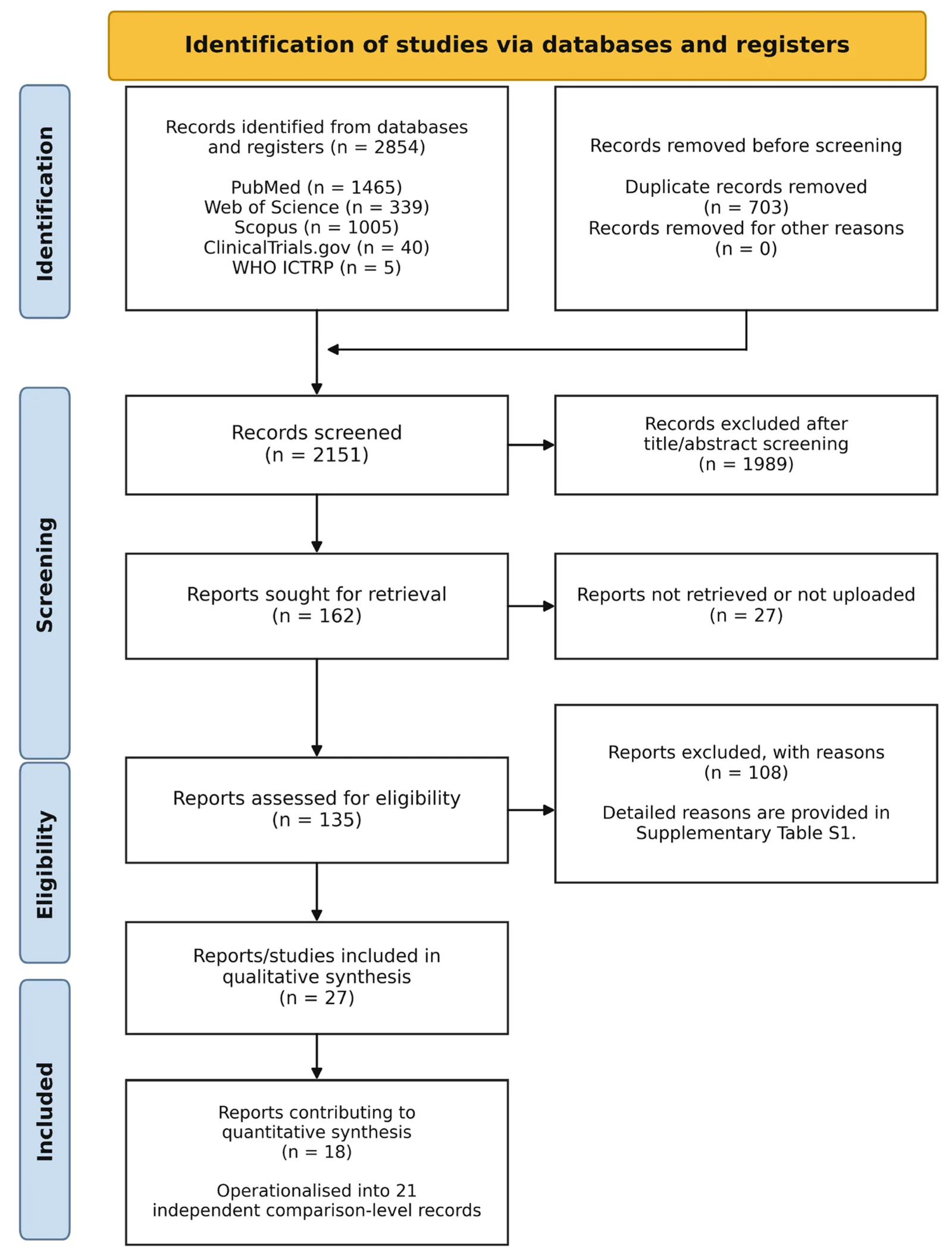
PRISMA 2020 flow diagram. After duplicate removal, 2151 records were screened, 135 reports were assessed, 27 reports/studies were retained for qualitative synthesis, and 18 reports were candidate sources for quantitative synthesis (21 independent comparison-level records). The 27 reports not assessed could not be evaluated because the full text was unavailable or had not been obtained for review.

The official search set identified 2854 records. After removal of 703 duplicates, 2151 unique records were screened. The full-text screening matrix was reconciled to 162 PRISMA-countable reports/records. Of these, 135 reports were assessed at full text or with sufficient available material, 27 reports could not be assessed because the full text was unavailable or had not been obtained for review, 108 were excluded after full-text assessment with standardized reasons, and 27 reports/studies were retained for qualitative synthesis. Eighteen reports were considered candidate sources for quantitative synthesis and were operationalized into 21 independent comparison-level records. The complete study-selection process is shown in Figure 1, and detailed reasons for full-text exclusion are provided in Supplementary Table S1.

### 3.2. Primary Total Testosterone Extraction Status

Total testosterone extraction was completed before synthesis. The clean final-value total testosterone dataset contains 11 independent comparisons suitable for the primary model. The primary dataset excludes Holt 2024 [33] because the placebo-arm final testosterone confidence interval was internally inconsistent and excludes Amini 2020 [29] because the reported testosterone unit requires verification. Amini 2020 [29] may be considered only in a sensitivity analysis assuming ng/mL; Holt 2024 [33] requires author clarification or corrected data. The 11 included total-testosterone comparisons were drawn from Gheflati et al. [27], Maghsoumi-Norouzabad et al. [28], Zittermann et al. [31], Pilz et al. [17], Saha et al. [20], Ulrich et al. [21], Rips et al. [22], Mielgo-Ayuso et al. [23], Michalczyk et al. [24], and Ramezani Ahmadi et al. [25]. The final-value total testosterone extraction dataset is summarized in Table 2.

### 3.3. Risk-of-Bias Results

Across the 11 total testosterone comparisons, one was judged at low risk of bias, eight as having some concerns, and two as high risk. The low-risk comparison was Maghsoumi-Norouzabad 2021 [28]. High-risk comparisons were Zittermann 2019 [31]/EVITA and Pilz 2011 [17]. The remaining comparisons were judged as having some concerns, mainly because testosterone was a secondary or exploratory outcome, because analyses were restricted to male subgroups, or because per-protocol/incomplete outcome data and cointerventions could influence the estimate. The domain-level RoB 2 judgements for final total testosterone are presented in Table 3.

### 3.4. Quantitative Synthesis of Total Testosterone

The primary random-effects model included 11 comparisons. Vitamin D supplementation was not associated with a statistically clear difference in final total testosterone concentrations compared with control (mean difference 0.47 nmol/L, 95% CI −0.50 to 1.44; *p* = 0.306). Between-study heterogeneity was low to moderate (tau^2^ = 0.360; *I*^2^ = 24.1%), and the prediction interval ranged from −1.21 to 2.15 nmol/L, indicating that effects in future similar studies may plausibly range from small decreases to small increases in total testosterone. The prespecified sensitivity analyses for final total testosterone concentrations are summarized in Table 4.

**Figure 2:**
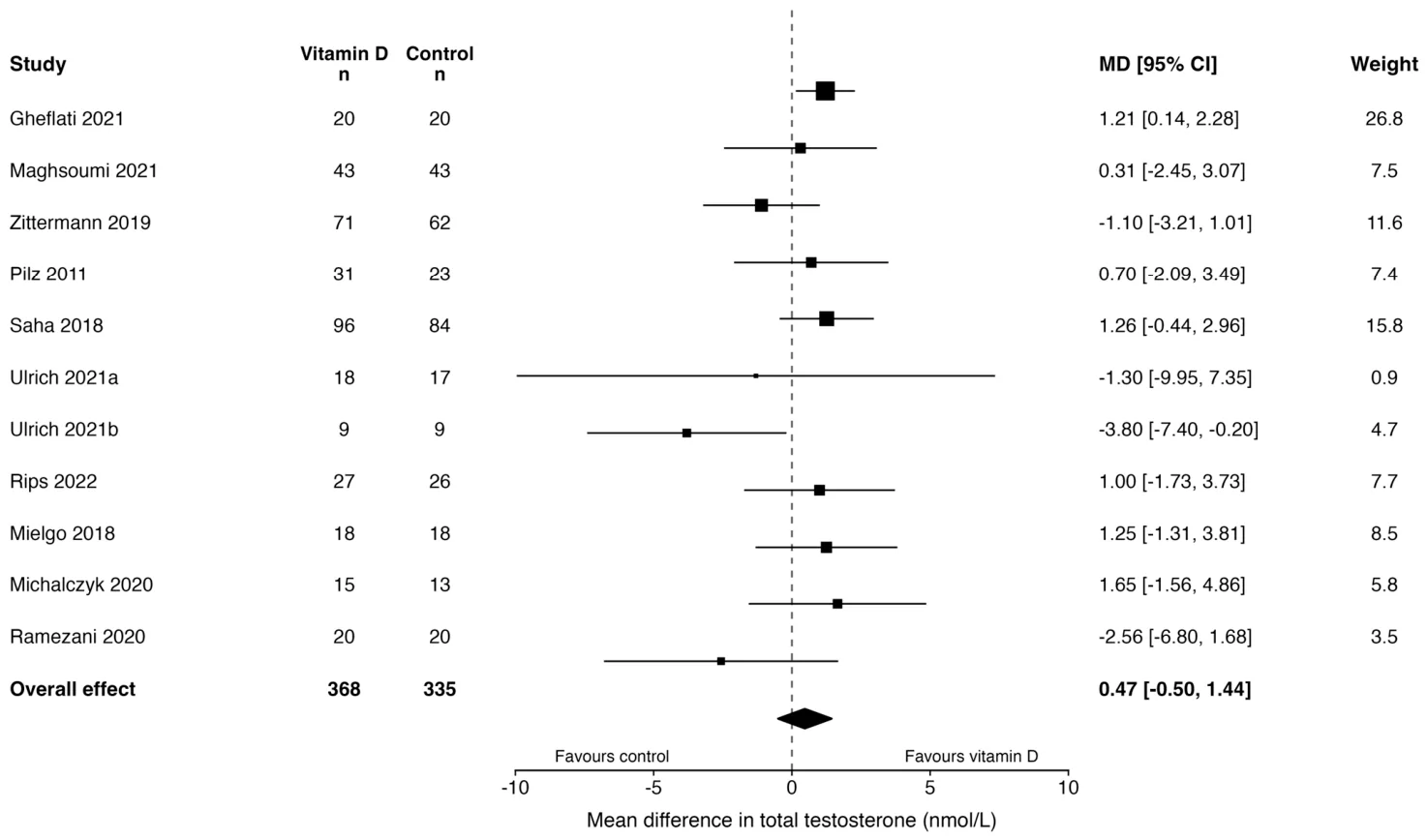
Forest plot of the effect of vitamin D supplementation on final total testosterone concentrations. Effect estimates are ex-pressed as mean differences in nmol/L. The dashed vertical line indicates the null effect (mean difference = 0). Solid horizontal lines represent the 95% confidence intervals for individual study estimates, and square markers represent the study-specific point estimates. The diamond represents the pooled effect estimate and its 95% confidence interval. The pooled estimate was calculated using a random-effects model with restricted maximum likelihood estimation and Hartung-Knapp adjustment. Positive values indicate higher final total testosterone concentrations in the vitamin D group compared with control. Study labels correspond to Gheflati et al. [27], Maghsoumi-Norouzabad et al. [28], Zittermann et al. [31], Pilz et al. [17], Saha et al. [20], Ulrich et al. [21], Rips et al. [22], Mielgo-Ayuso et al. [23], Michalczyk et al. [24], and Ramezani Ahmadi et al. [25].

The estimate remained directionally similar and not statistically clear after excluding high-risk-of-bias studies. The sensitivity analysis excluding clinically extreme populations produced a larger statistically significant estimate; however, this result should be interpreted as sensitivity-dependent and hypothesis-generating. It removed advanced heart failure and hemodialysis cohorts, thereby increasing clinical homogeneity but narrowing applicability. Excluding athlete, military, or active sport populations and excluding strong cointerventions attenuated the estimate toward the null. Therefore, the primary inference is based on the full prespecified TT model rather than on any single sensitivity analysis. The primary forest plot for final total testosterone concentrations is presented in Figure 2.

### 3.5. SHBG Extraction Status

SHBG final-value extraction was completed after source-level verification of the eligible full texts and tables. Seven comparisons were extractable. Five comparisons were eligible for the primary conservative model because they provided mean/SD data directly or allowed straightforward conversion from SE or 95% CI. Two additional comparisons from Lerchbaum 2017 [30] and Lerchbaum 2019 [18] were retained for sensitivity analysis because they required conversion from median and interquartile range. Gheflati 2021 [27] was retained with a unit-label flag because the table label was physiologically implausible, but the values were consistent with nmol/L. Saha 2018 [20] was analysed as a collapsed factorial vitamin D main-effect contrast. The SHBG final-value extraction dataset and conversion/status decisions are summarized in Table 5.

### 3.6. Quantitative Synthesis of SHBG

The primary conservative random-effects model included five comparisons. Vitamin D supplementation was not associated with a statistically clear difference in final SHBG concentrations compared with control (mean difference 0.27 nmol/L, 95% CI −2.14 to 2.68; *p* = 0.770). Between-study heterogeneity was not observed (tau^2^ = 0.000; *I*^2^ = 0.0%; Q = 5.85, *p* = 0.211), and the prediction interval ranged from −2.14 to 2.68 nmol/L.

**Figure 3:**
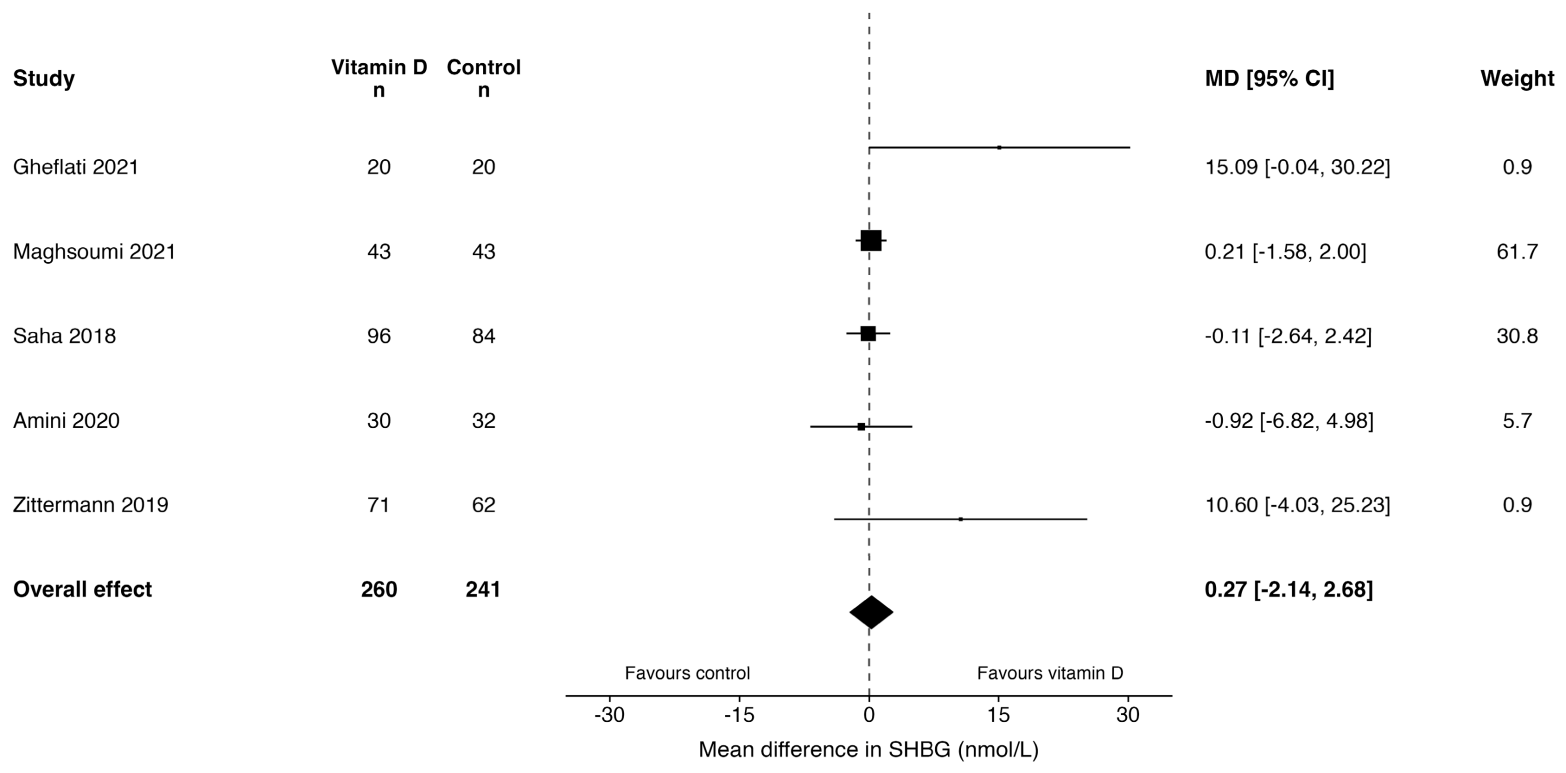
Forest plot of the effect of vitamin D supplementation on final sex hormone-binding globulin (SHBG) concentrations. Effect estimates are expressed as mean differences in nmol/L. The dashed vertical line indicates the null effect (mean difference = 0). Solid horizontal lines represent the 95% confidence intervals for individual study estimates, and square markers represent the study-specific point estimates. The diamond represents the pooled effect estimate and its 95% confidence interval. The pooled estimate was calculated using a random-effects model with restricted maximum likeli-hood estimation and Hartung-Knapp adjustment. Positive values indicate higher final SHBG concentrations in the vitamin D group compared with control. Study labels correspond to Gheflati et al. [27], Maghsoumi-Norouzabad et al. [28], Saha et al. [20], Amini et al. [29], and Zittermann et al. [31].

The sensitivity analysis including all seven extractable SHBG comparisons, including studies converted from medians and interquartile ranges, yielded a nearly identical null result (mean difference −0.14 nmol/L, 95% CI −2.38 to 2.09; *p* = 0.881). Heterogeneity remained absent (tau^2^ = 0.000; *I*^2^ = 0.0%; Q = 11.04, *p* = 0.087), and the prediction interval ranged from −2.38 to 2.09 nmol/L. These findings indicate that the SHBG result was robust to the inclusion of studies requiring statistical conversion. The primary and sensitivity analyses for final SHBG concentrations are summarized in Table 6. The primary SHBG forest plot is presented in Figure 3.

### 3.7. Free Androgen Index Extraction Status

FAI final-value extraction was completed after source-level verification of the eligible full texts and tables. Six comparisons were identified as potentially extractable. Three comparisons were retained for the primary conservative model because they reported FAI in a comparable scale with final mean and SD data. Lerchbaum 2019 [18] was retained for sensitivity analysis because values were reported as medians and interquartile ranges and converted to approximate means and SDs. Gheflati 2021 [27] and Lerchbaum 2017 [30] were excluded from the primary quantitative model be-cause their FAI scales were not sufficiently comparable for a defensible pooled estimate. The final-value FAI extraction dataset, scale-compatibility status, and analysis decisions are summarized in Table 7.

### 3.8. Quantitative Synthesis of Free Androgen Index

The primary conservative random-effects model included three comparisons with comparable FAI scaling. Vitamin D supplementation was not associated with a statistically clear difference in final FAI values compared with control (mean difference −0.37, 95% CI −4.28 to 3.55; *p* = 0.725). Between-study heterogeneity was not observed (tau^2^ = 0.000; *I*^2^ = 0.0%; Q = 0.52, *p* = 0.771), and the prediction interval ranged from −4.28 to 3.55.

**Figure 4:**
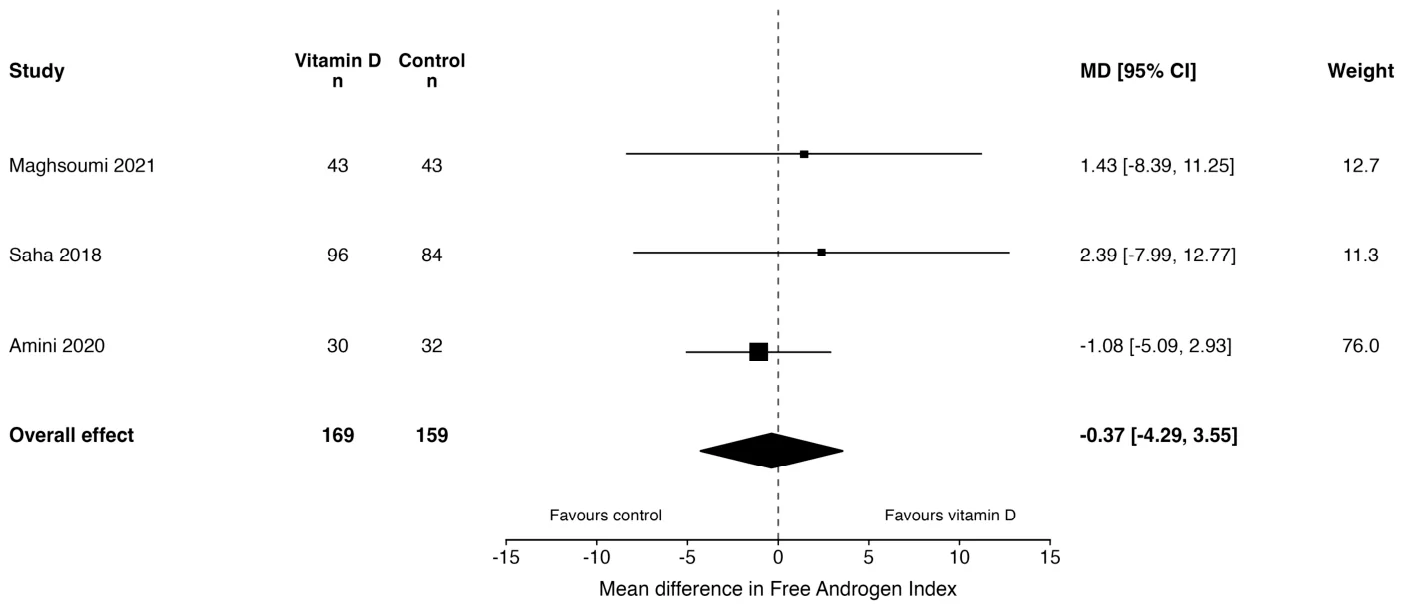
Forest plot of the effect of vitamin D supplementation on final free androgen index (FAI). Effect estimates are expressed as mean differences using comparable FAI scaling. The pooled estimate was calculated using a random-effects model with restricted maximum likelihood estimation and Hartung–Knapp adjustment. Positive values indicate higher final FAI values in the vitamin D group compared with control. Study labels correspond to Maghsoumi-Norouzabad et al. [28], Saha et al. [20] and Amini et al. [29].

A sensitivity analysis including Lerchbaum 2019 [18], converted from median and interquartile range, yielded a directionally higher but still non-significant pooled estimate (mean difference 2.35, 95% CI −4.57 to 9.27; *p* = 0.359). In this model, heterogeneity increased to a moderate level (tau^2^ = 10.96; *I*^2^ = 45.1%; Q = 5.23, *p* = 0.156), and the prediction interval was wide (−10.26 to 14.95). Overall, the FAI findings do not support a statistically clear effect of vitamin D supplementation on androgen bioavailability as estimated by FAI. The primary FAI forest plot is presented in Figure 4.

### 3.9. Calculated Free Testosterone Extraction Status

Free testosterone extraction was completed with method-specific classification. Directly measured FT and calculated FT were not pooled together. Amini 2020 [29] reported directly measured FT by ECLIA and was retained for narrative interpretation only. Lerchbaum 2019 [18], Zittermann 2019/EVITA [31], and Pilz 2011 [17] reported calculated FT using the Vermeulen framework and were eligible for a sensitivity-only quantitative synthesis. Lerchbaum 2017 [30] was not included because the reported IQR for FT was internally questionable and precluded reliable conversion. Gheflati 2021 [27], Maghsoumi-Norouzabad 2021 [28], Heijboer 2015 [19], and Saha 2018 [20] did not provide extractable final-value FT data suitable for this synthesis. Jain 2024 [26] reported a free/total testosterone ratio graphically within a vitamin D plus L-cysteine co-supplementation design and was retained only for narrative contextualization. The method-specific FT extraction status and quantitative decisions are summarized in Table 8.

### 3.10. Quantitative Synthesis of Calculated Free Testosterone

Calculated free testosterone was evaluated in a sensitivity-only analysis restricted to studies using compatible calculated FT methods. Three comparisons were included. Vitamin D supplementation did not significantly affect final calculated free testosterone values compared with control (mean difference −0.0096 nmol/L, 95% CI −0.0525 to 0.0332; *p* = 0.435). No between-study heterogeneity was observed (tau^2^ = 0.000; I^2^ = 0.0%; Q = 1.35, *p* = 0.508), and the prediction interval ranged from −0.0525 to 0.0332. The calculated FT sensitivity analysis is summarized in Table 9.

### 3.11. Bioactive Testosterone Extraction Status and Exploratory Synthesis

Bioactive testosterone was reported with sufficient extractable final-value data in two studies, Pilz 2011 [17] and Zittermann 2019/EVITA [31]. Both reported BAT as a calculated index. Jain 2024 [26] was not eligible for the BAT analysis because it focused on bioavailable 25-hydroxyvitamin D and reported an androgen-related free/total testosterone ratio within a vitamin D plus L-cysteine co-supplementation design rather than a compatible vitamin D-only BAT outcome. Heijboer 2015 [19] and Saha 2018 [20] did not report extractable BAT. The BAT extraction status and quantitative decisions are summarized in Table 10.

The exploratory BAT analysis did not show a statistically significant effect of vitamin D supplementation on final bioactive testosterone values (mean difference −0.47 nmol/L, 95% CI −1.77 to 0.83; *p* = 0.137). No heterogeneity was detected (tau^2^ = 0.000; I^2^ = 0.0%; Q = 0.07, *p* = 0.787), although this analysis should be interpreted cautiously because only two studies contributed data. The exploratory BAT analysis is summarized in Table 11.

### 3.12. GRADE Certainty of Evidence and Summary of Findings

The certainty of evidence was low for TT, SHBG, and FAI, and very low for calculated FT and BAT. Downgrading was mainly driven by outcome-level risk-of-bias concerns, imprecision, small numbers of comparisons for some outcomes, and the method-restricted or exploratory nature of calculated FT and BAT. Statistical inconsistency was not serious in the main models, but clinical and methodological heterogeneity was considered in interpretation. Detailed GRADE judgements are provided in Supplementary Table S5. The Summary of Findings and GRADE certainty ratings are presented in Table 12.

This systematic review and meta-analysis found no statistically clear or reproducible effect of vitamin D supplementation on TT or androgen bioavailability markers in adult men. Across the prespecified outcome hierarchy, pooled estimates for TT, SHBG, FAI, calculated FT, and BAT were compatible with no clear effect. The certainty of evidence was low for TT, SHBG, and FAI and very low for calculated FT and BAT. These findings should be interpreted as evidence that current randomized trials do not demonstrate a consistent androgen-related benefit of vitamin D supplementation, rather than as definitive proof that vitamin D has no biological relevance in all male endocrine contexts.

The present results are consistent with the broader vitamin D trial literature, where extra-skeletal effects often appear weaker in randomized evidence than in observational studies [3,4,5,6]. Vitamin D supplementation effects depend on baseline 25(OH)D status, dose, formulation, adherence, duration, body composition, and whether participants are deficient or already replete at baseline [3,4,5,6]. The androgen trials included here were heterogeneous across these dimensions, and most were not designed primarily to test androgen-related hypotheses. Therefore, absence of a pooled effect may reflect a true lack of clinically relevant androgenic action, insufficient targeting of biologically plausible subgroups, or limited precision of secondary biomarker reporting.

The randomized literature does not point in a single direction. Pilz et al. reported within-group increases in TT, calculated FT, and BAT after one year of vitamin D supplementation in overweight men participating in a weight-loss program [17]. However, subsequent trials were less supportive. Lerchbaum et al. found no significant effect on TT or secondary androgen outcomes in men with low baseline testosterone [18], Zittermann et al. reported no between-group differences in TT, SHBG, calculated FT, or BAT in men with advanced heart failure [31], and Heijboer et al., Jorde et al., and Ulrich et al. did not demonstrate a consistent testosterone-raising effect [19,21,32]. The positive signal from early work should therefore be regarded as hypothesis-generating rather than confirmatory.

Several features may explain these discrepancies. First, androgen outcomes were often secondary or post hoc biochemical measures rather than prespecified primary endpoints. Second, the trials enrolled clinically diverse populations, including infertile men, healthy men, overweight men, athletes, dialysis patients, and men with advanced heart failure. Third, cointerventions such as weight loss, athletic training, calcium co-supplementation, or prior sunlight exposure may have influenced endocrine markers independently of vitamin D. Fourth, biochemical methods varied across studies, including differences in TT, SHBG, 25(OH)D, FT, and BAT ascertainment. These issues support the conservative synthesis strategy used in this review.

The TT model provides the broadest quantitative anchor because TT was the most consistently extractable androgen outcome. The primary model showed a small pooled mean difference with a confidence interval crossing the null and a prediction interval compatible with small effects in either direction. Excluding high-risk-of-bias studies did not materially change the conclusion. The sensitivity analysis excluding clinically extreme populations became statistically significant, but it should not be overread: it was sensitivity-dependent, removed advanced heart failure and hemodialysis cohorts, and narrowed generalizability. Conversely, excluding athlete, military, active sport, or strong cointervention studies attenuated the estimate toward the null. Overall, the TT findings suggest that apparent signals are context-dependent and vulnerable to study-selection decisions.

SHBG and FAI are central to the androgen bioavailability framework. SHBG influences the interpretation of TT by determining the proportion of hormone that is free or weakly albumin-bound. In this review, vitamin D supplementation did not show a clear effect on SHBG, and the all-study sensitivity analysis produced a similar null result. FAI also did not show a clear effect in the conservative scale-compatible model. Although the FAI sensitivity analysis including a median/IQR-derived comparison shifted the estimate upward, the confidence interval remained wide. These findings do not support a consistent vitamin D-mediated effect through SHBG-related androgen distribution or calculated androgen index changes.

Free testosterone required particularly strict handling. Directly measured FT and calculated FT were not pooled because they are analytically non-equivalent and may differ in calibration, assay performance, assumptions, and dependence on TT, SHBG, and albumin [8,9,10]. The quantitative FT analysis was therefore restricted to method-compatible calculated FT studies, and it did not show a clear effect. BAT was even more limited, with compatible data available from only two studies; it was therefore treated as exploratory. This approach avoids producing misleading precision from biologically related but analytically non-equivalent biomarkers.

The present synthesis also helps contextualize observational associations between 25(OH)D and androgen markers. Such associations may reflect shared determinants rather than a direct causal pathway. Adiposity, insulin resistance, inflammation, physical activity, sunlight exposure, chronic disease, and general health status can influence both vitamin D status and androgen physiology. Modern mechanistic literature on Leydig-cell aging and metabolic dysfunction supports the biological plausibility of endocrine vulnerability, but it also indicates that testosterone decline is multifactorial and unlikely to be reversed by a single micronutrient intervention in unselected populations [13,14]. Randomized evidence therefore remains essential.

Compared with prior syntheses, this review adds several methodological refinements. We focused on randomized controlled trials, applied comparison-level data handling, used outcome-specific RoB 2 assessment, implemented GRADE certainty judgments, separated direct and calculated FT, restricted FAI pooling to compatible scales, and distinguished primary, sensitivity, and exploratory analyses. These decisions make the synthesis more conservative, but also more defensible in the literature where endocrine outcomes are frequently secondary, incompletely reported, or measured using heterogeneous biochemical methods.

This review has several strengths. It was prospectively registered, followed PRISMA 2020 and Cochrane methodological guidance, used structured extraction and audit trails, assessed risk of bias at the outcome level, and documented scale-compatibility and extractability decisions in the Supplementary Materials. The review also avoided combining analytically non-equivalent constructs, which is particularly important for FT and BAT.

Several limitations should be acknowledged. First, Embase was not searched because institutional access was unavailable. This deviation from the original plan was documented before screening, extraction, risk-of-bias assessment, and synthesis, but it remains a real limitation because eligible intervention studies may be variably indexed. Second, 27 reports could not be assessed because the full text was unavailable or had not been obtained for review. Third, several potentially relevant studies could not be included in quantitative synthesis because final values, dispersion measures, units, or scale definitions were unavailable, internally inconsistent, or not comparable. Fourth, the broad adult male framework improved feasibility but introduced clinical heterogeneity. Finally, most androgen outcomes were secondary or exploratory endpoints, limiting confidence in effect-modification analyses and formal publication-bias assessment.

Future trials should be designed specifically to test androgen-related hypotheses rather than treating testosterone and bioavailability markers as secondary observations. Priority should be given to men with confirmed vitamin D deficiency and biochemical hypogonadism, because this subgroup is the most biologically plausible population in which an endocrine effect, if present, might be detected. Trials should prespecify TT, SHBG, calculated FT, and BAT; use standardized morning sampling; report baseline and achieved 25(OH)D using traceable assays where possible; clearly describe vitamin D formulation, dose, frequency, adherence, and cointerventions; distinguish direct from calculated FT; report albumin when derived estimates are used; and provide complete final values with dispersion measures. Without these improvements, future evidence will remain difficult to pool and vulnerable to the same interpretive limitations identified here.

## 4. Conclusions

In summary, the available randomized evidence does not demonstrate a consistent effect of vitamin D supplementation on TT or androgen bioavailability markers in adult men, including SHBG, FAI, calculated FT, and BAT. The certainty of evidence was low for TT, SHBG, and FAI and very low for calculated FT and BAT. These findings should not be interpreted as definitive proof of absence of biological activity in all male subgroups; rather, they indicate that current randomized trials do not provide reproducible evidence of an androgen-related benefit. Future trials should focus on men with confirmed vitamin D deficiency and biochemical hypogonadism, prespecify androgen outcomes, standardize FT ascertainment, and report complete extractable data.

## Figures and Tables

**Table 1:** Conceptual and analytical interpretation of androgen-related outcomes used in this review.

Marker	Biological/Clinical Meaning	Main Methodological Issue	Role in This Review
Total testosterone	Total circulating testosterone, including bound and unbound fractions.	May not reflect androgen bioavailability when SHBG is altered.	Primary quantitative anchor because it was most consistently extractable.
SHBG	Carrier protein that influences the distribution and interpretation of circulating testosterone.	Changes may alter TT interpretation without necessarily reflecting increased androgen production.	Primary androgen bioavailability marker.
FAI	Calculated index generally derived from TT/SHBG × 100.	Scale definitions varied across trials; non-comparable scales should not be pooled.	Primary conservative model restricted to comparable scaling.
Free testosterone	Unbound fraction of testosterone.	Directly measured and calculated FT are analytically non-equivalent.	Calculated FT analyzed only as method-compatible sensitivity evidence.
Bioactive/bioavailable testosterone	Free plus weakly albumin-bound testosterone, usually calculated in contributing trials.	Few compatible studies and variable reporting.	Exploratory outcome only.

**Table 2:** Final-value total testosterone extraction dataset and outcome-specific RoB 2 overall judgement for the primary quantitative synthesis.

Study/Comparison	Population Category	n Int/Ctrl	Final TT Intervention	Final TT Control	RoB 2 Overall
Gheflati 2021 [27]	Infertility	20/20	4.61 ± 2.06	3.40 ± 1.30	Some concerns
Maghsoumi-Norouzabad 2021 [28]	Infertility	43/43	13.69 ± 6.07	13.38 ± 6.97	Low risk
Zittermann 2019 [31]	Advanced heart failure	71/62	10.00 ± 5.28	11.10 ± 6.89	High risk
Pilz 2011 [17]	Overweight/weight loss	31/23	13.40 ± 4.70	12.70 ± 5.50	High risk
Saha 2018 [20]	Healthy young men	96/84	19.81 ± 6.62	18.55 ± 5.02	Some concerns
Ulrich 2021a [21]	Healthy men	18/17	20.50 ± 7.90	21.80 ± 16.50	Some concerns
Ulrich 2021b [21]	Hemodialysis	9/9	7.80 ± 3.80	11.60 ± 4.00	Some concerns
Rips 2022 [22]	Military/physically active	27/26	21.30 ± 5.90	20.30 ± 4.10	Some concerns
Mielgo-Ayuso 2018 [23]	Elite athletes	18/18	16.40 ± 4.44	15.15 ± 3.33	Some concerns
Michalczyk 2020 [24]	Elite athletes	15/13	28.25 ± 3.20	26.60 ± 5.10	Some concerns
Ramezani Ahmadi 2020 [25]	Physically active men	20/20	18.24 ± 3.50	20.80 ± 9.01	Some concerns

Notes: Total testosterone values are final post-intervention values expressed in nmol/L. Saha 2018 [20] uses a collapsed cholecalciferol versus non-cholecalciferol factorial contrast. Ulrich 2021 [21] contributes two clinically distinct male comparisons. RoB 2 judgments are outcome-specific for final total testosterone.

**Table 3:** Outcome-specific RoB 2 domain-level judgements for final total testosterone.

Study/Comparison	D1	D2	D3	D4	D5	Overall
Gheflati 2021 [27]	Some concerns	Low risk	Some concerns	Low risk	Some concerns	Some concerns
Maghsoumi-Norouzabad 2021 [28]	Low risk	Low risk	Low risk	Low risk	Low risk/minor concerns	Low risk
Zittermann 2019 [31]	Low risk/minor concerns	Low risk	High risk	Some concerns	Low risk	High risk
Pilz 2011 [17]	Some concerns	Low risk/minor concerns	Some concerns	Low risk	High risk/serious concerns	High risk
Saha 2018 [20]	Low risk	Some concerns	Some concerns	Low risk	Some concerns	Some concerns
Ulrich 2021a [21]	Some concerns	Low risk	Some concerns	Low risk	Some concerns	Some concerns
Ulrich 2021b [21]	Low risk	Low risk	Some concerns	Low risk	Some concerns	Some concerns
Rips 2022 [22]	Low risk	Low risk	Some concerns	Low risk	Some concerns	Some concerns
Mielgo-Ayuso 2018 [23]	Some concerns	Some concerns	Low risk	Low risk	Some concerns	Some concerns
Michalczyk 2020 [24]	Some concerns	Some concerns	Some concerns	Low risk	Some concerns	Some concerns
Ramezani Ahmadi 2020 [25]	Low risk	Some concerns	Some concerns	Low risk	Some concerns	Some concerns

**Table 4:** Sensitivity analyses for final total testosterone concentrations.

Analysis	Exclusion Rule	k	MD (nmol/L)	95% CI	*p* Value	tau^2^	*I* ^2^
Primary analysis	All 11 primary TT comparisons	11	0.47	−0.50 to 1.44	0.306	0.360	24.1%
Excluding high-RoB studies	Zittermann 2019 [31] and Pilz 2011 [17] removed	9	0.84	−0.14 to 1.81	0.083	0.000	22.2%
Excluding clinically extreme populations	Advanced heart failure and hemodialysis removed	9	1.01	0.43 to 1.59	0.004	0.000	0.0%
Excluding athletes/military/active sport populations	Athlete, military, and active sport cohorts removed	7	0.21	−1.27 to 1.69	0.744	0.969	41.7%
Excluding strong cointerventions	Pilz 2011 [17], Saha 2018 [20], and Michalczyk 2020 [24] removed	8	−0.06	−1.52 to 1.41	0.931	1.195	41.7%

**Table 5:** Final-value SHBG extraction dataset and conversion/status decisions.

Study/Comparison	n Int/Ctrl	Final SHBG Intervention	Final SHBG Control	Conversion/Status
Gheflati 2021 [27]	20/20	47.82 ± 30.63	32.73 ± 15.92	SE to SD; nmol/L probable; unit-label flag
Maghsoumi-Norouzabad 2021 [28]	43/43	19.09 ± 3.69	18.88 ± 4.71	Direct mean ± SD
Saha 2018 [20]	96/84	21.11 ± 8.24	21.22 ± 8.97	Collapsed factorial contrast
Amini 2020 [29]	30/32	25.76 ± 9.99	26.68 ± 13.53	Direct mean ± SD; study-level unit caution
Zittermann 2019 [31]	71/62	49.00 ± 56.12	38.40 ± 26.52	95% CI to SD; wide CI caution
Lerchbaum 2019 [18]	46/47	28.53 ± 14.00	35.33 ± 15.26	Median/IQR to mean/SD; sensitivity
Lerchbaum 2017 [30]	48/49	38.70 ± 11.85	39.40 ± 13.70	Median/IQR to mean/SD; sensitivity

Notes: Values are final post-intervention SHBG concentrations expressed in nmol/L. Converted values are approximate and were used according to the prespecified primary-versus-sensitivity analysis structure. Saha 2018 [20] uses the collapsed cholecalciferol versus non-cholecalciferol factorial contrast.

**Table 6:** Primary and sensitivity analyses for final SHBG concentrations.

Analysis	Inclusion Rule	k	MD (nmol/L)	95% CI	*p* Value	tau^2^	*I* ^2^	Prediction Interval
Primary conservative analysis	Means/SDs direct or SE/CI-derived	5	0.27	−2.14 to 2.68	0.770	0.000	0.0%	−2.14 to 2.68
Sensitivity analysis, all studies	Adds median/IQR-derived studies	7	−0.14	−2.38 to 2.09	0.881	0.000	0.0%	−2.38 to 2.09

**Table 7:** Final-value Free Androgen Index extraction dataset, scale-compatibility status, and analysis decision.

Study/Comparison	n Int/Ctrl	Final FAI Intervention	Final FAI Control	Conversion/Status	Analysis Decision
Gheflati 2021 [27]	20/20	0.16 ± 0.13	0.26 ± 0.45	SE to SD; probable simple-ratio scale; not comparable with ×100 FAI	Excluded from primary; exploratory only
Maghsoumi-Norouzabad 2021 [28]	43/43	40.56 ± 22.71	39.13 ± 23.72	Direct mean ± SD; FAI = T/SHBG × 100 (%)	Primary conservative model
Saha 2018 [20]	96/84	101.61 ± 35.34	99.22 ± 35.53	Collapsed factorial contrast; FAI reported as TT/SHBG ratio	Primary conservative model
Amini 2020 [29]	30/32	14.66 ± 6.05	15.74 ± 9.74	Direct mean ± SD; FAI = TT/SHBG × 100; no recalculation from TT performed	Primary conservative model with caution
Lerchbaum 2019 [18]	46/47	46.70 ± 15.86	38.40 ± 18.53	Median/IQR to mean/SD; FAI = TT/SHBG × 100	Sensitivity analysis
Lerchbaum 2017 [30]	48/49	7.40 ± 3.04	6.80 ± 3.41	Median/IQR to mean/SD; reported formula × 100 but values indicate incompatible scale	Excluded from primary and main sensitivity

Notes: Values are final post-intervention FAI values. Saha 2018 [20] uses the collapsed cholecalciferol versus non-cholecalciferol factorial contrast. Converted values are approximate and were used only according to the prespecified primary-versus-sensitivity analysis structure. Amini 2020 [29] was analyzed using the author-reported FAI only; FAI was not recalculated from total testosterone because the testosterone unit required separate caution.

**Table 8:** Method-specific free testosterone extraction status and quantitative decision.

Study/Comparison	FT Extractable	Method	Quantitative Decision
Amini 2020 [29]	Yes	Direct FT, ECLIA	Narrative only; not pooled with calculated FT
Lerchbaum 2019 [18]	Yes	Calculated FT, Vermeulen	Sensitivity analysis
Zittermann 2019/EVITA [31]	Yes	Calculated FT, Vermeulen	Sensitivity analysis
Pilz 2011 [17]	Yes	Calculated FT, Vermeulen	Sensitivity analysis
Lerchbaum 2017 [30]	Partial	Calculated FT, Vermeulen	Excluded pending IQR verification
Gheflati 2021 [27]	No	Not reported	Not extractable
Maghsoumi-Norouzabad 2021 [28]	No	Not reported	Not extractable
Heijboer 2015 [19]	No	Only total testosterone	Not extractable for FT
Saha 2018 [20]	No	Not reported	Not extractable
Jain 2024 [26]	Ratio only	Free/total testosterone ratio; graphical; VD + L-cysteine design	Narrative only

Notes: FT = free testosterone. The quantitative FT model was restricted to calculated FT studies to avoid combining direct assay-based FT with equation-derived FT. Additional extraction details and decision rules are provided in Supplementary Table S3.

**Table 9:** Sensitivity analysis restricted to method-compatible calculated free testosterone.

Analysis	Inclusion Rule	k	MD (nmol/L)	95% CI	*p* Value	tau^2^	*I* ^2^	Prediction Interval
Calculated FT sensitivity	Vermeulen-calculated FT only	3	−0.0096	−0.0525 to 0.0332	0.435	0.000	0.0%	−0.0525 to 0.0332

**Table 10:** Bioactive testosterone extraction status and quantitative decision.

Study/Comparison	BAT Extractable	Method	Quantitative Decision
Pilz 2011 [17]	Yes	Calculated BAT	Exploratory analysis
Zittermann 2019/EVITA [31]	Yes	Calculated BAT	Exploratory analysis
Jain 2024 [26]	No	Bioavailable 25(OH)D; free/total testosterone ratio	Narrative only; not BAT
Heijboer 2015 [19]	No	Not reported	Not extractable
Saha 2018 [20]	No	Not reported	Not extractable

**Table 11:** Exploratory analysis for final bioactive testosterone concentrations.

Analysis	Inclusion Rule	k	MD (nmol/L)	95% CI	*p* Value	tau^2^	*I* ^2^	Prediction Interval
BAT exploratory	Calculated BAT studies only	2	−0.47	−1.77 to 0.83	0.137	0.000	0.0%	−1.77 to 0.83

**Table 12:** Summary of findings and certainty of evidence for vitamin D supplementation and androgen-related outcomes.

Outcome	Evidence Base	Effect Estimate	Certainty of Evidence	GRADE Downgrading Domains	Interpretation
Total testosterone	11 comparisons; 703 participants	MD 0.47 nmol/L; 95% CI −0.50 to 1.44	Low	Downgraded for risk of bias and imprecision. Inconsistency not serious; indirectness not serious for the broad adult male framework; publication bias not formally assessable.	Vitamin D supplementation did not show a clear effect on final TT concentrations.
Sex hormone-binding globulin	5 comparisons; 501 participants	MD 0.27 nmol/L; 95% CI −2.14 to 2.68	Low	Downgraded for risk of bias and imprecision. Inconsistency not serious; indirectness not serious; publication bias not formally assessable.	Vitamin D supplementation did not show a clear effect on final SHBG concentrations.
Free androgen index	3 comparisons; 328 participants	MD −0.37; 95% CI −4.29 to 3.55	Low	Downgraded for risk of bias and imprecision. Inconsistency not serious in the primary model; indirectness not serious for comparable FAI scaling; publication bias not formally assessable.	Vitamin D supplementation did not show a clear effect on final FAI.
Calculated free testosterone	3 comparisons; 279 participants	MD −0.010 nmol/L; 95% CI −0.053 to 0.033	Very low	Downgraded for risk of bias, serious imprecision, and indirectness because this was sensitivity-only evidence restricted to method-compatible calculated FT studies. Inconsistency not serious; publication bias not formally assessable.	A sensitivity analysis restricted to method-compatible calculated FT did not suggest a clear effect.
Bioactive testosterone	2 comparisons; 187 participants	MD −0.47 nmol/L; 95% CI −1.77 to 0.83	Very low	Downgraded for very serious imprecision and indirectness because this was exploratory evidence from only two studies. Risk-of-bias concerns were considered; inconsistency not estimable with confidence; publication bias not formally assessable.	Exploratory evidence did not suggest a clear effect, but the estimate remains highly uncertain.

Notes: CI, confidence interval; FAI, free androgen index; FT, free testosterone; MD, mean difference; SHBG, sex hormone-binding globulin. Certainty of evidence was assessed using the GRADE framework. Randomized controlled trials started as high-certainty evidence and were rated down for concerns related to risk of bias, inconsistency, indirectness, imprecision, and publication bias. Outcomes based on sensitivity-only or exploratory analyses were additionally downgraded for indirectness and limited evidence base.

## Data Availability

The original contributions presented in this study are included in the article and Supplementary Materials. The extraction audit, comparison-level mapping, risk-of-bias matrix, and GRADE details are provided in the Supplementary Materials. The analytic dataset and R code can be made available by the corresponding author upon reasonable request.

## Supplementary Materials

The following supporting information can be downloaded at https://www.mdpi.com/article/10.3390/nu18132090/s1, Supplementary File S1: Search strategies; Supplementary Table S1: Full-text exclusions with PRISMA reasons; Supplementary Table S2: Included studies and comparison-level mapping; Supplementary Table S3: Extraction audit for TT, SHBG, FAI, FT, and BAT; Supplementary Table S4: RoB 2 domain-level matrix with rationales; Supplementary Table S5: GRADE downgrading details by domain; Supplementary Figure S1: SHBG sensitivity forest plot; Supplementary Figure S2: FAI sensitivity forest plot; Supplementary Figure S3: calculated free testosterone sensitivity forest plot; Supplementary Figure S4: bioactive testosterone exploratory forest plot; Supplementary Figure S5: Summary of findings and GRADE certainty of evidence figure.

## References

[1] Holick M.F. (2007). Vitamin D deficiency. N. Engl. J. Med..

[2] Bikle D.D. (2014). Vitamin D metabolism, mechanism of action, and clinical applications. Chem. Biol..

[3] Giustina A., Bilezikian J.P., Adler R.A., Banfi G., Bikle D.D., Binkley N., Bollerslev J., Bouillon R., Brandi M.L., Casanueva F.F. (2024). Consensus statement on vitamin D status assessment and supplementation: Whys, whens, and hows. Endocr. Rev..

[4] Demay M.B., Pittas A.G., Bikle D.D., Diab D.L., Kiely M.E., Lazaretti-Castro M., Lips P., Mitchell D.M., Murad M.H., Powers S. (2024). Vitamin D for the prevention of disease: An Endocrine Society clinical practice guideline. J. Clin. Endocrinol. Metab..

[5] Bouillon R., Manousaki D., Rosen C., Trajanoska K., Rivadeneira F., Richards J.B. (2022). The health effects of vitamin D supplementation: Evidence from human studies. Nat. Rev. Endocrinol..

[6] Bendotti G., Biamonte E., Leporati P., Goglia U., Ruggeri R.M., Gallo M. (2025). Vitamin D supplementation: Practical advice in different clinical settings. Nutrients.

[7] Bhasin S., Brito J.P., Cunningham G.R., Hayes F.J., Hodis H.N., Matsumoto A.M., Snyder P.J., Swerdloff R.S., Wu F.C., Yialamas M.A. (2018). Testosterone therapy in men with hypogonadism: An Endocrine Society clinical practice guideline. J. Clin. Endocrinol. Metab..

[8] Vermeulen A., Verdonck L., Kaufman J.M. (1999). A critical evaluation of simple methods for the estimation of free testosterone in serum. J. Clin. Endocrinol. Metab..

[9] Jayasena C.N., Anderson R.A., Llahana S., Barth J., MacKenzie F., Wilkes S., Smith N., Sooriakumaran P., Minhas S., Wu F.C.W. (2022). Society for Endocrinology guidelines for testosterone replacement therapy in male hypogonadism. Clin. Endocrinol..

[10] Fiers T., Wu F., Moghetti P., Vanderschueren D., Lapauw B., Kaufman J.M. (2018). Reassessing free-testosterone calculation by liquid chromatography-tandem mass spectrometry direct equilibrium dialysis. J. Clin. Endocrinol. Metab..

[11] Blomberg Jensen M., Nielsen J.E., Jørgensen A., Rajpert-De Meyts E., Kristensen D.M., Jørgensen N., Skakkebaek N.E., Juul A., Leffers H. (2010). Vitamin D receptor and vitamin D metabolizing enzymes are expressed in the human male reproductive tract. Hum. Reprod..

[12] Blomberg Jensen M. (2012). Vitamin D metabolism, sex hormones, and male reproductive function. Reproduction.

[13] Kaltsas A., Dimitriadis F., Zachariou A., Sofikitis N. (2026). When testosterone fades: Leydig cell aging shaped by environmental toxicants, metabolic dysfunction, and testicular niche crosstalk. Cells.

[14] Luo D., Qi X., Xu X., Yang L., Yu C., Guan Q. (2023). Involvement of p38 MAPK in Leydig cell aging and age-related decline in testosterone. Front. Endocrinol..

[15] Lee D.M., Tajar A., Pye S.R., Boonen S., Vanderschueren D., Bouillon R., O’Neill T.W., Bartfai G., Casanueva F.F., Finn J.D. (2012). Association of hypogonadism with vitamin D status: The European Male Ageing Study. Eur. J. Endocrinol..

[16] Wehr E., Pilz S., Boehm B.O., März W., Obermayer-Pietsch B. (2010). Association of vitamin D status with serum androgen levels in men. Clin. Endocrinol..

[17] Pilz S., Frisch S., Koertke H., Kuhn J., Dreier J., Obermayer-Pietsch B., Wehr E., Zittermann A. (2011). Effect of vitamin D supplementation on testosterone levels in men. Horm. Metab. Res..

[18] Lerchbaum E., Trummer C., Theiler-Schwetz V., Kollmann M., Wölfler M., Heijboer A.C., Pilz S., Obermayer-Pietsch B. (2019). Effects of vitamin D supplementation on androgens in men with low testosterone levels: A randomized controlled trial. Eur. J. Nutr..

[19] Heijboer A.C., Oosterwerff M., Schroten N.F., Eekhoff E.M.W., Chel V.G.M., de Boer R.A., Blankenstein M.A., Lips P. (2015). Vitamin D supplementation and testosterone concentrations in male human subjects. Clin. Endocrinol..

[20] Saha S., Goswami R., Ramakrishnan L., Vishnubhatla S., Mahtab S., Kar P., Srinivasan S., Singh N., Singh U. (2018). Vitamin D and calcium supplementation, skeletal muscle strength and serum testosterone in young healthy adult males: Randomized control trial. Clin. Endocrinol..

[21] Ulrich C., Trojanowicz B., Fiedler R., Kraus F.B., Stangl G.I., Girndt M., Seibert E. (2021). Serum testosterone levels are not modified by vitamin D supplementation in dialysis patients and healthy subjects. Nephron.

[22] Rips L., Toom A., Kuik R., Varblane A., Mölder H., Tammaru M., Kull M., Ööpik V., Kartus J.-T., Gapeyeva H. (2022). Seven-month wintertime supplementation of 1200 IU vitamin D has no effect on hand grip strength in young, physically active males: A randomized, controlled study. J. Int. Soc. Sports Nutr..

[23] Mielgo-Ayuso J., Calleja-González J., Urdampilleta A., León-Guereño P., Córdova A., Caballero-García A., Fernandez-Lázaro D. (2018). Effects of vitamin D supplementation on haematological values and muscle recovery in elite male traditional rowers. Nutrients.

[24] Michalczyk M.M., Gołaś A., Maszczyk A., Kaczka P., Zając A. (2020). Influence of sunlight and oral D3 supplementation on serum 25(OH)D concentration and exercise performance in elite soccer players. Nutrients.

[25] Ramezani Ahmadi A., Mohammadshahi M., Alizadeh A., Ahmadi Angali K., Jahanshahi A. (2020). Effects of vitamin D3 supplementation for 12 weeks on serum levels of anabolic hormones, anaerobic power, and aerobic performance in active male subjects: A randomized, double-blind, placebo-controlled trial. Eur. J. Sport Sci..

[26] Jain S.K., Justin Margret J., Zachary A., Lally M.M., Vanchiere J.A., Mhanna M.J., Shi R., Levine S.N. (2024). Effects of vitamin D and L-cysteine cosupplementation on circulating bioavailable and total 25-hydroxy-vitamin D, the free/total testosterone ratio and inflammatory biomarkers in healthy vitamin D-deficient African Americans: A placebo-controlled double-blind clinical trial. BMJ Nutr. Prev. Health.

[27] Gheflati A., Mirjalili S.A.M., Kaviani M., Salehi-Abargouei A., Hosseini-Marnani E., Nadjarzadeh A. (2021). Effects of vitamin D supplementation on semen quality and reproductive hormones in patients with asthenozoospermia: A randomized double-blind placebo-controlled clinical trial. J. Nutr. Food Secur..

[28] Maghsoumi-Norouzabad L., Zare Javid A., Mansoori A., Dadfar M., Serajian A. (2021). The effects of Vitamin D3 supplementation on Spermatogram and endocrine factors in asthenozoospermia infertile men: A randomized, triple blind, placebo-controlled clinical trial. Reprod. Biol. Endocrinol..

[29] Amini L., Mohammadbeigi R., Vafa M., Haghani H., Vahedian-Azimi A., Karimi L., Jahanfar S., Jamialahmadi T., Talebi A., Sahebkar A. (2020). Evaluation of the effect of vitamin D3 supplementation on quantitative and qualitative parameters of spermograms and hormones in infertile men: A randomized controlled trial. Complement. Ther. Med..

[30] Lerchbaum E., Pilz S., Trummer C., Schwetz V., Pachernegg O., Heijboer A.C., Obermayer-Pietsch B. (2017). Vitamin D and testosterone in healthy men: A randomized controlled trial. J. Clin. Endocrinol. Metab..

[31] Zittermann A., Ernst J.B., Prokop S., Fuchs U., Dreier J., Kuhn J., Knabbe C., Berthold H.K., Gouni-Berthold I., Gummert J.F. (2019). Vitamin D supplementation does not prevent the testosterone decline in males with advanced heart failure: The EVITA trial. Eur. J. Nutr..

[32] Jorde R., Grimnes G., Hutchinson M.S., Kjærgaard M., Kamycheva E., Svartberg J. (2013). Supplementation with vitamin D does not increase serum testosterone levels in healthy males. Horm. Metab. Res..

[33] Holt R., Yahyavi S.K., Kooij I., Poulsen N.N., Juul A., Jørgensen N., Jensen M.B. (2024). Effects of vitamin D on sex steroids, luteinizing hormone, and testosterone to luteinizing hormone ratio in 307 infertile men. Andrology.

[34] Abu-Zaid A., Saleh S.A.K., Adly H.M., Baradwan S., Alharran A.H., Alkhaldi M.G., Alzayed M.M., Alotaibi M.N., Saad A.R., Alfayadh H.M. (2024). The impact of vitamin D on androgens and anabolic steroids among adult males: A meta-analytic review. Diseases.

[35] Binkley N., Sempos C.T., Vitamin D Standardization Program (2014). Standardizing vitamin D assays: The way forward. J. Bone Miner. Res..

[36] Wise S.A., Phinney K.W., Tai S.S.C., Camara J.E., Myers G.L., Durazo-Arvizu R., Tian L., Hoofnagle A.N., Bachmann L.M., Young I.S. (2017). Baseline assessment of 25-hydroxyvitamin D assay performance: A Vitamin D Standardization Program interlaboratory comparison study. J. AOAC Int..

[37] Page M.J., McKenzie J.E., Bossuyt P.M., Boutron I., Hoffmann T.C., Mulrow C.D., Shamseer L., Tetzlaff J.M., Akl E.A., Brennan S.E. (2021). The PRISMA 2020 statement: An updated guideline for reporting systematic reviews. BMJ.

[38] Higgins J.P.T., Thomas J., Chandler J., Cumpston M., Li T., Page M.J., Welch V.A. (2024). Cochrane Handbook for Systematic Reviews of Interventions, Version 6.5 (Updated August 2024).

[39] Sterne J.A.C., Savović J., Page M.J., Elbers R.G., Blencowe N.S., Boutron I., Cates C.J., Cheng H.Y., Corbett M.S., Eldridge S.M. (2019). RoB 2: A revised tool for assessing risk of bias in randomised trials. BMJ.

[40] Schünemann H., Brożek J., Guyatt G., Oxman A. (2013). GRADE Handbook for Grading Quality of Evidence and Strength of Recommendations Using the GRADE Approach.

