# Supplementary material for: Vitamin D Supplementation, Total Testosterone, and Androgen Bioavailability Markers in Adult Men: A Systematic Review and Meta-Analysis of Randomized Controlled Trials
Source: Nutrients. 2026 Jun 26;18(13):2090. doi: 10.3390/nu18132090 (PMC13363579; doi:10.3390/nu18132090)
Supplement: Supplementary file 1 [file nutrients-18-02090-s001.zip › nutrients-4355977-supplementary.pdf]

Supplementary Material

Vitamin D Supplementation, Total Testosterone, and Androgen Bioavailability Markers in Adult Men: A Systematic Review and Meta-Analysis of Randomized Controlled Trials

This supplementary file consolidates the search strategies, PRISMA full-text exclusion audit, included-study and comparison mapping, extraction audit, RoB 2 domain-level matrix with rationales, GRADE downgrading details, and supplementary figures supporting the manuscript.

Supplementary File S1. Search strategies

Vitamin D Supplementation, Total Testosterone, and Androgen Bioavailability Markers in Adult Men: A Systematic Review and Meta-Analysis of Randomized Controlled Trials

Overview

Search period and scope. Searches were conducted from database inception to April 2026. The final validated search set used for PRISMA identification included PubMed, Web of Science/Science Citation Index, Scopus, ClinicalTrials.gov, and WHO ICTRP. Search strategies combined terms related to vitamin D exposure or supplementation with terms related to testosterone, sex hormone-binding globulin, Free Androgen Index, free testosterone, bioavailable/bioactive testosterone, and androgen bioavailability.

Protocol amendment. Embase was initially planned as a bibliographic source but was not searched because institutional access was unavailable. This amendment was made before title/abstract screening, data extraction, risk-of-bias assessment, and synthesis, and is reported in the main manuscript and PRISMA documentation.

Search structure. For PubMed, Web of Science, and Scopus, a broad PICO strategy was complemented by a targeted strategy focused on hypogonadism/testosterone deficiency terminology. Trial registers were searched using shorter syntax adapted to platform capabilities.

Validated PRISMA identification counts

Full database and register search strategies

PubMed - main search (broad PICO)

(("Vitamin D"[Mesh] OR "Cholecalciferol"[Mesh] OR "Ergocalciferols"[Mesh] OR "Dietary Supplements"[Mesh] OR vitamin D[tiab] OR vitamin D3[tiab] OR vitamin D2[tiab] OR cholecalciferol[tiab] OR ergocalciferol[tiab] OR calcifediol[tiab] OR calcidiol[tiab] OR "25-hydroxyvitamin D"[tiab] OR "25(OH)D"[tiab]) AND ("Testosterone"[Mesh] OR "Androgens"[Mesh] OR "Sex Hormone-Binding Globulin"[Mesh] OR testosterone[tiab] OR "free testosterone"[tiab] OR "bioavailable testosterone"[tiab] OR "free androgen index"[tiab] OR FAI[tiab] OR SHBG[tiab] OR "sex hormone-binding globulin"[tiab] OR "sex hormone binding globulin"[tiab] OR androgens[tiab] OR "androgen bioavailability"[tiab] OR "androgen status"[tiab]) AND ("Male"[Mesh] OR "Adult"[Mesh] OR male[tiab] OR men[tiab] OR man[tiab] OR adult men[tiab]) AND (randomized controlled trial[pt] OR controlled clinical trial[pt] OR random*[tiab] OR placebo[tiab] OR trial[tiab] OR "double blind"[tiab] OR "double-blind"[tiab])) NOT (animals[mh] NOT humans[mh])

PubMed - complementary targeted search (hypogonadism/testosterone deficiency)

(("Vitamin D"[Mesh] OR "Cholecalciferol"[Mesh] OR "Ergocalciferols"[Mesh] OR vitamin D[tiab] OR vitamin D3[tiab] OR vitamin D2[tiab] OR cholecalciferol[tiab] OR ergocalciferol[tiab] OR calcifediol[tiab] OR "25-hydroxyvitamin D"[tiab] OR "25(OH)D"[tiab]) AND ("Testosterone"[Mesh] OR "Androgens"[Mesh] OR "Sex Hormone-Binding Globulin"[Mesh] OR "free testosterone"[tiab] OR "bioavailable testosterone"[tiab] OR "free androgen index"[tiab] OR SHBG[tiab] OR "sex hormone-binding globulin"[tiab] OR "androgen bioavailability"[tiab]) AND ("Hypogonadism"[Mesh] OR hypogonadism[tiab] OR "male hypogonadism"[tiab] OR "testosterone deficiency"[tiab] OR "low testosterone"[tiab] OR "androgen deficiency"[tiab] OR "late-onset hypogonadism"[tiab] OR "functional hypogonadism"[tiab]) AND ("Male"[Mesh] OR male[tiab] OR men[tiab]) AND (randomized controlled trial[pt] OR controlled clinical trial[pt] OR random*[tiab] OR placebo[tiab] OR trial[tiab])) NOT (animals[mh] NOT humans[mh])

Web of Science / Science Citation Index - main search (broad PICO)

TS=(("vitamin D" OR "vitamin D3" OR "vitamin D2" OR cholecalciferol OR ergocalciferol OR calcifediol OR calcidiol OR "25-hydroxyvitamin D" OR "25(OH)D") AND (testosterone OR "free testosterone" OR "bioavailable testosterone" OR "free androgen index" OR FAI OR SHBG OR "sex hormone-binding globulin" OR "sex hormone binding globulin" OR androgens OR "androgen bioavailability" OR "androgen status") AND (male OR males OR men OR man OR "adult men") AND (random* OR placebo* OR trial OR "double blind" OR "double-blind"))

Web of Science / Science Citation Index - complementary targeted search

TS=(("vitamin D" OR "vitamin D3" OR "vitamin D2" OR cholecalciferol OR ergocalciferol OR calcifediol OR "25-hydroxyvitamin D" OR "25(OH)D") AND ("free testosterone" OR "bioavailable testosterone" OR "free androgen index" OR SHBG OR "sex hormone-binding globulin" OR "androgen bioavailability" OR testosterone) AND (hypogonadism OR "male hypogonadism" OR "testosterone deficiency" OR "low testosterone" OR "androgen deficiency" OR "late-onset hypogonadism" OR "functional hypogonadism") AND (male OR men) AND (random* OR placebo* OR trial))

Scopus - main search (broad PICO)

TITLE-ABS-KEY(("vitamin D" OR "vitamin D3" OR "vitamin D2" OR cholecalciferol OR ergocalciferol OR calcifediol OR calcidiol OR "25-hydroxyvitamin D" OR "25(OH)D") AND (testosterone OR "free testosterone" OR "bioavailable testosterone" OR "free androgen index" OR FAI OR SHBG OR "sex hormone-binding globulin" OR "sex hormone binding globulin" OR androgens OR "androgen bioavailability" OR "androgen status") AND (male OR males OR men OR man OR "adult men") AND (random* OR placebo* OR trial OR "double blind" OR "double-blind"))

Scopus - complementary targeted search

TITLE-ABS-KEY(("vitamin D" OR "vitamin D3" OR "vitamin D2" OR cholecalciferol OR ergocalciferol OR calcifediol OR "25-hydroxyvitamin D" OR "25(OH)D") AND ("free testosterone" OR "bioavailable testosterone" OR "free androgen index" OR SHBG OR "sex hormone-binding globulin" OR "androgen bioavailability" OR testosterone) AND (hypogonadism OR "male hypogonadism" OR "testosterone deficiency" OR "low testosterone" OR "androgen deficiency" OR "late-onset hypogonadism" OR "functional hypogonadism") AND (male OR men) AND (random* OR placebo* OR trial))

ClinicalTrials.gov

("vitamin D" OR cholecalciferol OR ergocalciferol) AND (testosterone OR "free testosterone" OR SHBG OR "sex hormone-binding globulin" OR "free androgen index")

WHO ICTRP

("vitamin D" OR cholecalciferol OR ergocalciferol) AND (testosterone OR "free testosterone" OR SHBG OR "sex hormone-binding globulin" OR "androgen bioavailability")

Notes for reproducibility

The validated PRISMA counts shown above correspond to the final record set used in the manuscript and PRISMA flow diagram.

Duplicate removal was performed in Zotero before title/abstract screening.

ClinicalTrials.gov and WHO ICTRP syntax was adapted to the shorter Boolean formats accepted by each register interface.

No Embase search was conducted because institutional access was unavailable; this deviation from the initial plan was documented as a protocol amendment before screening and synthesis.

Complete retrieved records, screening decisions, and reasons for exclusion are documented in the main manuscript and supplementary tables.

Source	Validated PRISMA count
PubMed	1465
Web of Science / Science Citation Index	339
Scopus	1005
ClinicalTrials.gov	40
WHO ICTRP	5
Total records identified	2854

Supplementary Table S1. Full-text excluded reports and reasons (PRISMA 2020)

Supplementary Table S1. Full-text excluded reports and reasons (PRISMA 2020) — Summary

Supplementary Table S1. Full-text excluded reports and reasons (PRISMA 2020)	
PRISMA-reconciled full-text universe	162
Reports not retrieved or not uploaded	27
Reports assessed for eligibility	135
Reports excluded after full-text assessment	108
Reports/studies retained for qualitative synthesis	27
Scope note	This supplementary table lists the 108 reports excluded after eligibility assessment. Reports not retrieved or not uploaded are counted in PRISMA as not retrieved and are not included as excluded full-text reports.
Counting rule	Report-level table. Trial registries and overlapping/duplicate reports are retained for traceability when screened but are not double-counted as independent studies in synthesis.
Exclusion reason counts	
PRISMA exclusion reason	n
Review/non-original article	21
Wrong intervention / no eligible androgen outcome	7
Animal/preclinical study	6
Review/non-original article / wrong intervention	6
Review/non-original article / wrong outcome	6
No vitamin D supplementation/intervention	5
Wrong intervention	5
Non-human study / wrong intervention / wrong outcome	4
Wrong intervention / no eligible vitamin D comparator	4
Editorial/commentary	3
Wrong population / no eligible androgen outcome	3
Wrong population / wrong intervention / no eligible androgen outcome	3
Case report / wrong intervention	2

Supplementary Table S1. Full-text excluded reports and reasons (PRISMA 2020)	
Case report / wrong intervention / confounded cointervention	2
Commentary / wrong outcome	2
Duplicate/overlapping report	2
No eligible androgen outcome	2
Review/non-original article / wrong population	2
Wrong intervention / confounded cointervention	2
Case report / no eligible vitamin D intervention on androgen outcome	1
Confounded cointervention / no eligible comparator	1
Editorial/commentary / wrong intervention	1
Editorial/commentary / wrong outcome	1
Letter/commentary / no eligible androgen outcome	1
Mixed population without male-specific data	1
No male-specific data	1
No vitamin D supplementation as intervention / no eligible androgen outcome	1
No vitamin D supplementation/intervention / no eligible androgen outcome	1
Non-human study / wrong intervention	1
Opinion/editorial / wrong outcome	1
Trial registry / duplicate-overlapping report	1
Trial registry / duplicate-overlapping report / wrong intervention	1
Wrong intervention / confounded cointervention / wrong population	1
Wrong intervention / mixed population without male-specific data	1
Wrong intervention / no eligible comparator	1
Wrong intervention / wrong population / no eligible androgen outcome	1
Wrong intervention / wrong population / observational exposure	1
Wrong outcome / wrong intervention / no eligible androgen outcome	1

**Supplementary Table S1. Full-text excluded reports and reasons (PRISMA 2020)**	
Wrong population	1
Wrong study design	1

Supplementary Table S1. Full-text excluded reports and reasons (PRISMA 2020) — Excluded reports

ID	Year	Article title / report	Standardized PRISMA reason for exclusion	Methodological note
1	2026	Vitamin D Adequacy Conditions the Prolactin-Suppressive Effect of Metformin in Men Receiving Prolactin-Elevating Medications	Wrong intervention	Intervention was metformin, not vitamin D supplementation.
2	2026	Spatial localization and pharmacodynamic assessment of intracrine androgen metabolism in CRPC xenograft tumor tissues upon active vitamin D treatment	Animal/preclinical study	Non-human tumor/xenograft model.
3	2025	Light physical activity and vitamin D improve sperm quality of male Rattus norvegicus exposed to cigarette smoke	Animal/preclinical study	Non-human study; both groups received vitamin D.
4	2025	Vitamin D in Endocrine Disorders: A Broad Overview of Evidence in Musculoskeletal, Thyroid, Parathyroid, and Reproductive Disorders	Review/non-original article	May be used for background/citation chasing only.
5	2025	Therapeutic advances in sarcopenia management: From traditional interventions to personalized medicine	Review/non-original article	No original eligible data.
6	2025	Exposure to Aldehydes, Heterocyclic Aromatic Amines and Terpenes With Sex Hormones in U.S. Children and Adolescents: Sex-, Age-Dependent Patterns and Vitamin D Effect Modification	Wrong population	Pediatric/adolescent population; no vitamin D supplementation.
7	2025	Circulating growth hormone, cortisol and testosterone in relation to vitamin D status: influence of lower and upper body Wingate Anaerobic Test in elite artistic gymnasts	No vitamin D supplementation/intervention	Vitamin D is status variable, not intervention.
8	2025	Sheer drop ahead: reviewing sarcopenia outcomes in elderly patients undergoing bariatric surgery	Review/non-original article	No primary eligible data.
9	2025	The Effects of Vitamin D on Muscle Strength Are Influenced by Testosterone Levels	Wrong study design	Human component observational; intervention preclinical.
11	2024	The Impact of Vitamin D on Androgens and Anabolic Steroids among Adult Males: A Meta-Analytic Review	Review/non-original article	Use for reference tracking only.
12	2024	Effects of high-intensity statin therapy on steroid hormones and vitamin D in type 2 diabetic men	Wrong intervention	Vitamin D measured as biomarker, not intervention.
17	2023	Low serum anti-Müllerian hormone is associated with semen quality in infertile men and not influenced by vitamin D supplementation	Duplicate/overlapping report	Use ID 13 as primary report for androgen outcomes.

ID	Year	Article title / report	Standardized PRISMA reason for exclusion	Methodological note
19	2023	Effects of Gender and Vitamin D on Vascular Reactivity of the Carotid Artery on a Testosterone-Induced PCOS Model	Animal/preclinical study	Non-human study.
21	2023	Dietary cholecalciferol and 25-hydroxycholecalciferol supplementation interact to modulate reproductive performance, egg quality, serum antioxidant capacity, intestinal morphology and tibia quality of breeder geese	Animal/preclinical study	Non-human.
22	2023	Effect of vitamin D3 supplementation in winter on physical performance of university students	No male-specific data	No male-specific hormonal data extractable.
24	2022	The effect of vitamin D3 administration and its combination with physical activity on oxidative stress markers in old rats	Animal/preclinical study	Non-human.
25	2022	Functional hypogonadism among patients with obesity, diabetes, and metabolic syndrome	Review/non-original article	No original data.
26	2022	Effects of a multicomponent resistance-based exercise program with protein, vitamin D and calcium supplementation on cognition in men with prostate cancer treated with ADT	Wrong intervention / no eligible androgen outcome	Multicomponent; vitamin D not isolated; both groups received vitamin D.
27	2022	Biomarker Changes in Response to a 12-Week Supplementation of an Oral Nutritional Supplement Enriched with Protein, Vitamin D and HMB	Wrong intervention / mixed population without male-specific data	Multicomponent and no male-specific androgen data.
28	2022	Overlapping sperm damages from vitamin B or D deficiency in mice	Animal/preclinical study	Non-human.
30	2022	Vitamin D receptor and estrogen receptor gene polymorphisms in men with type 2 diabetes	No vitamin D supplementation/intervention	No supplementation.
32	2021	Musculoskeletal Responses to Exercise Plus Nutrition in Men with Prostate Cancer on Androgen Deprivation	Wrong intervention / no eligible androgen outcome	Multicomponent; both groups received vitamin D.
34	2021	Effects of hormonal changes on sarcopenia in chronic kidney disease	Review/non-original article	No primary data.
35	2021	Effects of high-dose vitamin D supplementation on phase angle and physical function in patients with prostate cancer on ADT	No eligible androgen outcome	Outcome is phase angle/physical function.
36	2021	Administration of Anabolic Steroids in Combination with Vitamin D in the Early Stage of Treatment of Polytrauma Patients	Wrong intervention	Anabolic steroid contaminates androgen axis.
38	2021	Cardiometabolic Risk Factors in Rosuvastatin-Treated Men with Mixed Dyslipidemia and Early-Onset Androgenic Alopecia	Wrong intervention	Vitamin D measured only.
45	2020	A bone to pick with vitamin D deficiency and erectile dysfunction	Editorial/commentary	No primary data.

ID	Year	Article title / report	Standardized PRISMA reason for exclusion	Methodological note
49	2020	Effects of Seasonal Vitamin D3 Supplementation on Strength, Power, and Body Composition in College Swimmers	Mixed population without male-specific data	Hormonal data not male-specific.
50	2020	Reviewing the Evidence on Vitamin D Supplementation in the Management of Testosterone Status and Its Effects on Male Reproductive System	Review/non-original article	Reference tracking only.
51	2020	The Combination of a Diversified Intake of Carbohydrates and Fats and Supplementation of Vitamin D in a Diet Does Not Affect Hormone Levels in Men Practicing Strength Training	Wrong intervention / no eligible comparator	All groups received vitamin D; macronutrient comparison.
52	2019	Sarcopenia associated with chemotherapy and targeted agents for cancer therapy	Review/non-original article	No eligible primary data.
53	2019	Bone and hormonal status 10 years post-allogeneic bone marrow transplantation	No vitamin D supplementation/intervention	Status assessment only.
54	2019	The effect of vitamin D on thyroid autoimmunity in euthyroid men with autoimmune thyroiditis and testosterone deficiency	Confounded cointervention / no eligible comparator	Testosterone cointervention and no placebo/no vitamin D comparator.
57	2018	A Prospective, Observational Study of Osteoporosis in Men	Wrong intervention / confounded cointervention	No isolated vitamin D effect.
61	2017	Possible influence of vitamin D on male reproduction	Review/non-original article	No primary data.
62	2017	Switch to restoration therapy in a testosterone treated central hypogonadism with erythrocytosis	Case report / wrong intervention	Not vitamin D intervention.
64	2017	Effect of testosterone replacement therapy on vitamin D and FGF-23 levels in congenital hypogonadism	Wrong intervention	Reverse causal direction.
66	2017	Hormones and Sarcopenia	Review/non-original article	Context only.
67	2017	Association between vitamin D and sperm parameters: Clinical evidence	No vitamin D supplementation/intervention	Observational association with 25(OH)D; no supplementation.
68	2017	The role of vitamin D in male fertility: A focus on the testis	Review/non-original article	Mechanistic review.
69	2016	Sarcopenia in heart failure: mechanisms and therapeutic strategies	Review/non-original article	No primary eligible data.
70	2016	Vitamin D and Polycystic Ovary Syndrome (PCOS): a review	Review/non-original article / wrong population	Female PCOS review.
71	2016	Testosterone replacement in 49,XXXXY syndrome: andrological, metabolic and neurological aspects	Case report / wrong intervention	Testosterone replacement; vitamin D supportive.
72	2016	Are low levels of 25(OH) vitamin D and testosterone clinically relevant in men with paraplegia?	Editorial/commentary	No primary data.
73	2015	Estrogen and bone health in men and women	Review/non-original article	Bone/sex steroids review.

ID	Year	Article title / report	Standardized PRISMA reason for exclusion	Methodological note
75	2015	Effect of Vitamin D Supplementation on Glycemic Control in Patients With Type 2 Diabetes — SUNNY Trial: Response to Comment	Letter/commentary / no eligible androgen outcome	Authors indicate testosterone was not measured.
78	2014	Multiple Hormonal Dysregulation as Determinant of Low Physical Performance and Mobility in Older Persons	Review/non-original article	Review on hormonal dysregulation.
79	2014	Effect of risedronate in osteoporotic HIV males, according to gonadal status: a pilot study	Wrong intervention / no eligible androgen outcome	Risedronate study; vitamin D/calcium co-supplementation.
80	2014	Androgen-deprivation-associated bone disease	Review/non-original article / wrong intervention	ADT bone disease review.
81	2013	Is androgen therapy indicated in men with osteoporosis?	Review/non-original article / wrong intervention	Androgen therapy review.
83	2013	Optimizing Bone Health and Minimizing Skeletal Morbidity in Men with Prostate Cancer	Review/non-original article / wrong intervention	Bone health in prostate cancer.
84	2013	Scientific overview of hormone treatment used for rejuvenation	Review/non-original article	No primary data.
85	2013	Androgen deprivation treatment in prostate cancer	Review/non-original article / wrong intervention	ADT review; vitamin D supportive only.
86	2012	Effects of vitamin D supplementation on androgens in men with low testosterone levels	Duplicate/overlapping report	Duplicate of ID55.
87	2012	Fracture Risk and Zoledronic Acid Therapy in Men with Osteoporosis	Wrong intervention / no eligible androgen outcome	Zoledronic acid RCT; vitamin D/calcium co-supplementation.
88	2012	The Challenges of Using Vitamin D in Cancer Prevention and Prognosis	Editorial/commentary	No primary eligible data.
89	2012	Calcium and Vitamin D Supplementation During Androgen Deprivation Therapy for Prostate Cancer: A Critical Review	Review/non-original article / wrong outcome	Calcium/vitamin D for bone under ADT.
90	2012	Effect of various drug treatments on bone density in hypogonadal men	Wrong intervention / confounded cointervention	Testosterone and bone drugs; vitamin D supportive.
91	2012	Hormonal Therapy of Intrinsic Aging	Review/non-original article	Review.
92	2011	Vitamin D Supplementation and Male Infertility: The Copenhagen Bone-Gonadal Study	Trial registry / duplicate-overlapping report	Registry for Copenhagen trial represented by ID13.
93	2011	Osteoporosis in Men: Insights for the Clinician	Review/non-original article	Review.
95	2011	Should frailty be treated with testosterone?	Editorial/commentary / wrong intervention	Testosterone therapy focus.
97	2010	Osteoporosis in patients with prostate cancer on long-term androgen deprivation therapy	Commentary / wrong outcome	No eligible androgen outcome.
98	2010	Bone health in the prostate cancer patient receiving androgen deprivation therapy	Review/non-original article / wrong outcome	ADT bone health review.
99	2010	CHARGE syndrome as unusual cause of hypogonadism	Case report / wrong intervention / confounded cointervention	Testosterone depot + bone agents.

ID	Year	Article title / report	Standardized PRISMA reason for exclusion	Methodological note
100	2010	Older Men’s Knowledge of Osteoporosis and the Prevalence of Risk Factors	No vitamin D supplementation/intervention / no eligible androgen outcome	Survey/cross-sectional.
101	2010	Bone complications of androgen deprivation therapy: screening, prevention, and treatment	Review/non-original article / wrong outcome	Review.
102	2010	Sex Hormones and Colorectal Cancer: What Have We Learned So Far?	Editorial/commentary / wrong outcome	Editorial.
103	2009	Prostate cancer: Androgen deprivation therapy and bone loss	Commentary / wrong outcome	Commentary.
104	2009	Low serum 25-hydroxyvitamin D concentrations in healthy young males	No vitamin D supplementation/intervention	Observational vitamin D status only.
105	2009	Dehydroepiandrosterone replacement therapy in older adults: 1- and 2-y effects on bone	Wrong intervention / no eligible vitamin D comparator	DHEA RCT; all received calcium/vitamin D.
106	2008	Randomized, Double-Blinded Phase II Evaluation of Docetaxel with or without Doxercalciferol in Metastatic Androgen-Independent Prostate Cancer	Wrong population / wrong intervention / no eligible androgen outcome	Oncologic doxercalciferol + chemotherapy.
107	2008	Intermittent Chemotherapy in Patients With Metastatic Androgen-Independent Prostate Cancer — ASCENT	Wrong population / wrong intervention / no eligible androgen outcome	High-dose calcitriol used as oncologic agent.
108	2008	Additive benefit of higher testosterone levels and vitamin D plus calcium supplementation in regard to fall risk reduction among older men and women	No eligible androgen outcome	Testosterone baseline predictor; fall risk outcome.
109	2007	Effect of Once-Weekly Oral Alendronate on Bone Loss in Men Receiving Androgen Deprivation Therapy for Prostate Cancer	Wrong intervention / no eligible androgen outcome	Alendronate RCT; all received calcium/vitamin D.
110	2007	Preservation of bone health in prostate cancer	Review/non-original article / wrong outcome	Review.
111	2007	Lifestyle Factors and Duration of Androgen Deprivation Affect Bone Mineral Density of Patients with Prostate Cancer During First Year of Therapy	No vitamin D supplementation as intervention / no eligible androgen outcome	BMD risk factors; supplement as lifestyle variable.
112	2006	Management of Side Effects of Androgen Deprivation Therapy	Review/non-original article / wrong outcome	Review.
113	2006	Preventing skeletal complications in androgen deprived men with prostate cancer: Time for action	Opinion/editorial / wrong outcome	Opinion.
114	2006	Pamidronate Reduces Bone Loss after Allogeneic Stem Cell Transplantation	Wrong intervention / wrong population / no eligible androgen outcome	Pamidronate; calcitriol/calcium background.
117	2005	Osteoporosis and low-trauma fracture in men	Review/non-original article	Review.
118	2005	Prevention of Bone Loss after Allogeneic Stem Cell Transplantation by Calcium, Vitamin D, and Sex Hormone Replacement with or without Pamidronate	Wrong intervention / confounded cointervention / wrong population	Calcium/vitamin D + sex hormone replacement; ± pamidronate.
119	2005	Preventing Bone Loss During Androgen Deprivation Therapy for Prostate Cancer: Early Experience with Neridronate	Wrong intervention / no eligible androgen outcome	Neridronate and ADT; vitamin D/calcium support.

ID	Year	Article title / report	Standardized PRISMA reason for exclusion	Methodological note
120	2005	Osteoporosis Associated with Excess Glucocorticoids	Review/non-original article	Review.
121	2005	Administration of 1α-OH vitamin D3 and calcium prevents bone mass loss in patients with advanced prostatic carcinoma after orchidectomy treated with complete androgenic blockade	Wrong population / no eligible androgen outcome	Bone mass after orchidectomy/blockade; no androgen outcome.
122	2004	High-Dose Calcitriol and Carboplatin in Metastatic Androgen-Independent Prostate Cancer	Wrong population / wrong intervention / no eligible androgen outcome	Oncologic calcitriol.
123	2004	Neridronate Prevents Bone Loss in Patients Receiving Androgen Deprivation Therapy for Prostate Cancer	Wrong intervention / no eligible androgen outcome	Neridronate; vitamin D/calcium support.
125	2003	Development of weekly high-dose calcitriol based therapy for prostate cancer	Review/non-original article / wrong intervention	Oncologic therapy review.
126	2003	Exploring the effects of luteinizing hormone-releasing hormone agonist therapy on bone health	Review/non-original article / wrong outcome	ADT bone health.
127	2002	Kallmann’s syndrome: skeletal and psychological aspects of late diagnosis	Case report / wrong intervention / confounded cointervention	hCG + vitamin D/calcium; testosterone due to hCG.
128	2002	Bone Mass, Bone Turnover, Vitamin D, and Estrogen Receptor Gene Polymorphisms in Male-to-Female Transsexuals	Wrong intervention / wrong population / observational exposure	Estrogen/antiandrogen exposure; no vitamin D intervention.
129	2002	The effects of three-month intravenous ibandronate on bone mineral density and bone remodeling in Klinefelter’s syndrome	Wrong intervention / no eligible vitamin D comparator	Ibandronate/calcium; vitamin D correction not androgen outcome.
130	2001	Prevention of Osteoporosis in Men With Prostate Cancer	Trial registry / duplicate-overlapping report / wrong intervention	Registry for alendronate/ADT context.
131	2001	Effects of Transdermal Testosterone on Cognitive Function and Health Perception in Older Men With Low Bioavailable Testosterone Levels	Wrong intervention / no eligible vitamin D comparator	Testosterone transdermal RCT; all received calcium/vitamin D.
132	2001	The role of tacrolimus-based immunosuppression on bone mineral density and bone turnover after cardiac transplantation: randomized trial with calcitriol	Wrong population / no eligible androgen outcome	Calcitriol for post-transplant bone outcomes.
133	2001	Androgens and male aging: current evidence of safety and efficacy	Review/non-original article / wrong intervention	Review of testosterone therapy.
134	2001	Effects of Transdermal Testosterone on Bone and Muscle in Older Men With Low Bioavailable Testosterone Levels	Wrong intervention / no eligible vitamin D comparator	Testosterone RCT; vitamin D/calcium background.
136	2000	Die Osteoporose des Mannes / Osteoporosis in Males	Review/non-original article	Review.
139	1999	A case of femoral neck fracture in a patient with severe testosterone deficiency	Case report / no eligible vitamin D intervention on androgen outcome	Case with 1α(OH)D3/calcitonin; no vitamin D effect on testosterone.
140	1999	Prevention of osteoporosis after cardiac transplantation: randomized double-blind trial with calcitriol	Wrong population / no eligible androgen outcome	Calcitriol for BMD after cardiac transplantation.

ID	Year	Article title / report	Standardized PRISMA reason for exclusion	Methodological note
141	1998	Sex Steroid Treatment of Constitutionally Tall Stature	Review/non-original article / wrong population	Pediatric/tall stature sex steroid review.
142	1997	The Metabolism of Vitamin D3 in Response to Testosterone	Non-human study / wrong intervention	Immature male chicks; testosterone modifies vitamin D metabolism.
143	1997	Glucocorticoid-Induced Osteoporosis: Evaluation, Prevention, and Treatment	Review/non-original article	Clinical review.
154	1968	Estrogenic antagonism of bone-resorptive action of vitamin D	Wrong outcome / wrong intervention / no eligible androgen outcome	High-dose vitamin D/calcium metabolism; hormonal therapy antagonizes bone resorption.
156	1964	Prevention by Methyltestosterone of Parathyroid Cyst Formation	Non-human study / wrong intervention / wrong outcome	Rats; methyltestosterone protects against DHT/calcium toxicity.
157	1963	A progeria-like syndrome produced by dihydrotachysterol and its prevention by methyltestosterone and ferric dextran	Non-human study / wrong intervention / wrong outcome	Female rats; DHT toxicity model.
158	1963	Effect of anabolic hormones and ferric dextran upon the progeria-like syndrome produced by dihydrotachysterol	Non-human study / wrong intervention / wrong outcome	Rats; methyltestosterone as protective agent.
159	1963	Effect of Vitamin E and Methyltestosterone upon the progeria-like syndrome produced by dihydrotachysterol	Non-human study / wrong intervention / wrong outcome	Rats; vitamin E/methyltestosterone against DHT toxicity.

Supplementary Table S1. Full-text excluded reports and reasons (PRISMA 2020) — Notes

Field	Description
Purpose	Report-level list of studies/reports excluded after full-text eligibility assessment, with a standardized PRISMA-compatible reason.
Counting rule	This table is report-level. It supports the PRISMA 2020 flow diagram box: Reports excluded, with reasons (n = 108).
Not retrieved records	The 27 reports not retrieved or not uploaded are counted separately in the PRISMA flow and are not included in this excluded-reports table.
Operational correction applied	Two rows outside the reconciled full-text universe (IDs 163 and 164) were removed from the uploaded draft table so that S1 aligns with the validated PRISMA counts: 162 sought, 27 not retrieved, 135 assessed, 108 excluded, 27 included.
Source	Corrected full-text screening matrix for vitamin D and androgen bioavailability review, PRISMA 2020 aligned.

Supplementary Table S2. Included studies and comparison-level mapping

Supplementary Table S2. Included studies and comparison-level mapping — Summary

Item	Count / status	Methodological note
Reports/studies retained for qualitative synthesis	27	Matches PRISMA flow and manuscript text.
Reports contributing to quantitative synthesis	18	Operationalised into independent comparison-level records.
Independent comparison-level records	21	Created only when male-specific, population-specific, or source-trial-specific data required separate analytic handling.
Primary total testosterone final-value comparisons	11	Used in the main random-effects REML + Hartung-Knapp model.
Primary SHBG comparisons	5	Primary conservative model; sensitivity model includes 7 comparisons.
Primary FAI comparisons	3	Primary conservative model; sensitivity model includes Lerchbaum 2019.
Calculated FT comparisons	3	Method-restricted sensitivity analysis; direct FT not pooled.
BAT comparisons	2	Exploratory analysis only.
Unit-of-analysis rule	Report-level to comparison-level	Multi-population reports were split only when clinically distinct male-specific datasets were reported; factorial arms were collapsed to avoid double counting.

Supplementary Table S2. Included studies and comparison-level mapping — Included reports

Report ID	Report label	Year	Reference status	Qualitative synthesis	Quantitative report	Comparison-level records	Mapping note	Main role in review
R01	Gheflati 2021	2021	Final reference reconciled	Yes	Yes	1	Single parallel RCT in vitamin D-deficient infertile men with asthenozoospermia.	TT, SHBG, FAI extraction; FAI scale not pooled in primary.
R02	Maghsoumi-Norouzabad 2021	2021	Final reference reconciled	Yes	Yes	1	Single parallel triple-blind RCT in infertile men with asthenozoospermia.	TT, SHBG, FAI primary data.
R03	Zittermann 2019 / EVITA	2019	Final reference reconciled	Yes	Yes	1	Advanced heart failure male subgroup; final data available for several androgen markers.	TT primary; SHBG sensitivity; calculated FT and BAT models.
R04	Pilz 2011	2011	Final reference reconciled	Yes	Yes	1	Male subgroup nested in weight-reduction trial; judged high RoB for TT.	TT primary; calculated FT and BAT models.

Report ID	Report label	Year	Reference status	Qualitative synthesis	Quantitative report	Comparison-level records	Mapping note	Main role in review
R05	Saha 2018	2018	Final reference reconciled	Yes	Yes	1	Two-by-two factorial trial collapsed to cholecalciferol vs no cholecalciferol contrast.	TT, SHBG and FAI after factorial arm collapse.
R06	Ulrich 2021	2021	Final reference reconciled	Yes	Yes	2	One report split into two clinically distinct comparisons: healthy men and hemodialysis patients.	TT primary model as two comparisons.
R07	Rips 2022	2022	Final reference reconciled	Yes	Yes	1	Young physically active/military-like male cohort.	TT primary model.
R08	Mielgo-Ayuso 2018	2018	Final reference reconciled	Yes	Yes	1	Elite male traditional rowers; athletic population sensitivity flag.	TT primary model.
R09	Michalczyk 2020	2020	Final reference reconciled	Yes	Yes	1	Elite soccer players; sunlight/oral D3 exposure context and sport population.	TT primary model; cointervention/sport sensitivity flag.
R10	Ramezani Ahmadi 2020	2020	Final reference reconciled	Yes	Yes	1	Active male subjects; exercise-performance context.	TT primary model.
R11	Amini 2020	2020	Final reference reconciled	Yes	Yes	1	Infertile men; TT unit caution. Author-reported FAI retained; direct FT narrative only.	SHBG and FAI primary-with-caution; direct FT narrative only; TT not in primary.
R12	Lerchbaum 2017	2017	Final reference reconciled	Yes	Yes	1	Healthy men; median/IQR data and scale issues for some outcomes.	SHBG sensitivity; FAI excluded for scale; FT excluded due to unreliable IQR conversion.
R13	Lerchbaum 2019	2019	Final reference reconciled	Yes	Yes	1	Men with low testosterone; values often reported as median/IQR.	SHBG sensitivity, FAI sensitivity, calculated FT sensitivity.
R14	Heijboer 2015	2015	Final reference reconciled	Yes	Yes	3	Post hoc analysis of three source trials; kept as separate source-trial comparisons, but not	Structured quantitative/narrative handling; no final-

Report ID	Report label	Year	Reference status	Qualitative synthesis	Quantitative report	Comparison-level records	Mapping note	Main role in review
							directly meta-analysable for final-value TT.	value TT meta-analysis.
R15	Jorde 2013	2013	Final reference reconciled	Yes	Yes	1	Healthy male trial with change-score information but no final SD for primary final-value TT model.	Change-score/narrative sensitivity; not primary final-value meta-analysis.
R16	Holt 2024	2024	Final reference reconciled	Yes	Yes	1	Infertile men; excluded from primary TT because of internally inconsistent placebo-arm final TT confidence interval.	Qualitative and extraction audit; requires author clarification for primary synthesis.
R17	Jain 2024	2024	Final reference reconciled	Yes	Yes	1	Vitamin D plus L-cysteine co-supplementation; reports free/total testosterone ratio graphically rather than compatible FT/BAT data.	Narrative contextualisation; not pooled in FT/BAT.
R18	Holt 2020	2020	Zotero record identified; retained for traceability	Yes	Yes	1	Report on sex steroid production in men with normal or impaired Leydig cell function; retained for traceability of candidate quantitative evidence.	Traceability record; not used in primary pooled outcome models.
R19	Maghsoumi-Norouzabad 2021 (Nutrition Journal companion report)	2021	Zotero record identified; overlap/companion report	Yes	No	0	Companion report from the same asthenozoospermia research programme; retained for qualitative traceability, not counted as an independent quantitative comparison.	Qualitative/traceability only.
R20	Lerchbaum 2019 (Nutrients body composition/metabolic report)	2019	Zotero record identified; overlap/companion report	Yes	No	0	Companion/overlapping report from vitamin D supplementation in men; not counted as a separate quantitative androgen comparison.	Qualitative/traceability only.
R21	Holt 2023	2023	Zotero record identified	Yes	No	0	Related infertile-men report on AMH/semen quality; used for context/traceability where applicable.	Qualitative/traceability only.

Report ID	Report label	Year	Reference status	Qualitative synthesis	Quantitative report	Comparison-level records	Mapping note	Main role in review
R22	Ööpik 2017	2017	Zotero record identified	Yes	No	0	Military training/vitamin D-related male cohort; retained for context but not compatible androgen meta-analysis.	Qualitative/traceability only.
R23	Timpmann 2024	2024	Zotero record identified	Yes	No	0	Conscript/vitamin D-related performance report; no compatible androgen endpoint for current quantitative synthesis.	Qualitative/traceability only.
R24	Savolainen 2024	2024	Zotero record identified	Yes	No	0	Vitamin D supplementation/exercise-related male cohort; no compatible androgen endpoint for current quantitative synthesis.	Qualitative/traceability only.
R25	Trummer 2018	2018	Zotero record identified	Yes	No	0	Systematic review/background source retained for citation tracking and contextualisation, not quantitative synthesis.	Citation tracking/context only.
R26	Hosseini Marnani 2019	2019	Zotero record identified	Yes	No	0	Previous systematic review/meta-analysis retained for citation tracking and comparison with prior evidence.	Citation tracking/context only.
R27	Abu-Zaid 2024	2024	Final reference reconciled	Yes	No	0	Recent meta-analytic review retained as contextual prior synthesis, not as primary evidence.	Citation tracking/context only.

Supplementary Table S2. Included studies and comparison-level mapping — Comparison mapping

Comparison ID	Report ID	Comparison label	Source report	Year	Population/context	Design	Intervention/comparator	Split/collapse rationale	Eligible androgen outcomes	Quantitative role	Methodological note
C01	R01	Gheflati 2021	Gheflati 2021	2021	Infertile men with asthenozoospermia	Parallel RCT	Vitamin D vs placebo	No split	TT; SHBG; FAI	TT primary; SHBG primary; FAI excluded from primary scale-compatible model	FAI reported on probable simple-ratio scale; not comparable with ×100 FAI.

Comparison ID	Report ID	Comparison label	Source report	Year	Population/context	Design	Intervention/comparator	Split/collapse rationale	Eligible androgen outcomes	Quantitative role	Methodological note
C02	R02	Maghsoumi-Norouzabad 2021	Maghsoumi-Norouzabad 2021	2021	Infertile men with asthenozoospermia	Parallel triple-blind RCT	Vitamin D3 vs placebo	No split	TT; SHBG; FAI	TT primary; SHBG primary; FAI primary	Low RoB for TT.
C03	R03	Zittermann 2019 / EVITA	Zittermann 2019	2019	Men with advanced heart failure	RCT / EVITA trial	Vitamin D3 vs placebo	No split	TT; SHBG; calculated FT; BAT	TT primary; SHBG primary/sensitivity; calculated FT sensitivity; BAT exploratory	High RoB for TT driven by missing outcome data.
C04	R04	Pilz 2011	Pilz 2011	2011	Overweight men in weight-reduction programme	RCT male subgroup	Vitamin D3 vs placebo	No split	TT; calculated FT; BAT	TT primary; calculated FT sensitivity; BAT exploratory	High RoB for TT; cointervention/weight-loss context.
C05	R05	Saha 2018 collapsed cholecalciferol contrast	Saha 2018	2018	Young healthy vitamin D-deficient men	Two-by-two factorial RCT	Cholecalciferol-containing arms vs non-cholecalciferol arms	Factorial arms collapsed to avoid double counting	TT; SHBG; FAI	TT primary; SHBG primary; FAI primary	Collapsed contrast documented; calcium cointervention handled by main-effect contrast.
C06	R06	Ulrich 2021a healthy men	Ulrich 2021	2021	Healthy men	RCT / cohort-specific subgroup	Vitamin D vs control	Split because healthy men reported separately	TT	TT primary	Clinically distinct from dialysis cohort.
C07	R06	Ulrich 2021b hemodialysis	Ulrich 2021	2021	Men on hemodialysis	RCT / cohort-specific subgroup	Vitamin D vs control	Split because hemodialysis cohort reported separately	TT	TT primary	Clinically extreme population sensitivity flag.
C08	R07	Rips 2022	Rips 2022	2022	Young physically active men	RCT	Vitamin D vs placebo	No split	TT	TT primary	Athlete/military/active sport sensitivity flag.
C09	R08	Mielgo-Ayuso 2018	Mielgo-Ayuso 2018	2018	Elite male traditional rowers	RCT	Vitamin D vs placebo/control	No split	TT	TT primary	Athlete sensitivity flag.
C10	R09	Michalczyk 2020	Michalczyk 2020	2020	Elite soccer players	RCT / sunlight and D3 context	Vitamin D3/sunlight exposure vs comparator	No split	TT	TT primary	Sport and cointervention sensitivity flag.
C11	R10	Ramezani Ahmadi 2020	Ramezani Ahmadi 2020	2020	Active male subjects	RCT	Vitamin D3 vs placebo	No split	TT	TT primary	Active sport sensitivity flag.
C12	R11	Amini 2020	Amini 2020	2020	Infertile men with abnormal spermogram parameters	Parallel RCT	Vitamin D3 vs placebo	No split	TT; SHBG; FAI; direct FT	SHBG primary; FAI primary with caution; direct FT narrative; TT excluded from primary	TT unit requires verification; use author-reported FAI only.
C13	R12	Lerchbaum 2017	Lerchbaum 2017	2017	Healthy men	RCT	Vitamin D vs placebo	No split	SHBG; FAI; calculated FT	SHBG sensitivity; FAI excluded; FT excluded due to unreliable	FAI scale inconsistent; FT IQR internally questionable.

Comparison ID	Report ID	Comparison label	Source report	Year	Population/context	Design	Intervention/comparator	Split/collapse rationale	Eligible androgen outcomes	Quantitative role	Methodological note
										IQR conversion	
C14	R13	Lerchbaum 2019	Lerchbaum 2019	2019	Men with low testosterone	RCT	Vitamin D vs placebo	No split	SHBG; FAI; calculated FT	SHBG sensitivity; FAI sensitivity; calculated FT sensitivity	Median/IQR converted to approximate mean/SD where defensible.
C15	R14	Heijboer 2015 source trial 1	Heijboer 2015	2015	Male participants from source trial 1	Post hoc analysis of source RCT	Vitamin D vs control	Kept separate by source trial	TT	Narrative/change-score sensitivity; not final-value primary	Final-value TT not directly meta-analysable; median/IQR/change data limitation.
C16	R14	Heijboer 2015 source trial 2	Heijboer 2015	2015	Male participants from source trial 2	Post hoc analysis of source RCT	Vitamin D vs control	Kept separate by source trial	TT	Narrative/change-score sensitivity; not final-value primary	Final-value TT not directly meta-analysable; median/IQR/change data limitation.
C17	R14	Heijboer 2015 source trial 3	Heijboer 2015	2015	Male participants from source trial 3	Post hoc analysis of source RCT	Vitamin D vs control	Kept separate by source trial	TT	Narrative/change-score sensitivity; not final-value primary	Final-value TT not directly meta-analysable; median/IQR/change data limitation.
C18	R15	Jorde 2013	Jorde 2013	2013	Healthy men	RCT	Vitamin D vs placebo	No split	TT	Change-score/narrative sensitivity; not final-value primary	Final SDs not reported for primary final-value model.
C19	R16	Holt 2024	Holt 2024	2024	Infertile men	RCT	Vitamin D vs placebo/control	No split	TT; sex steroids; LH; T/LH ratio	Qualitative/extraction audit; not primary TT	Placebo-arm final TT CI internally inconsistent; author clarification required.
C20	R17	Jain 2024	Jain 2024	2024	Vitamin D-deficient African Americans	Placebo-controlled double-blind clinical trial	Vitamin D and L-cysteine co-supplementation design	No split for current review	Free/total testosterone ratio	Narrative only; not pooled	Graphical ratio and VD+L-cysteine design not compatible with vitamin D-only FT/BAT pooling.
C21	R18	Holt 2020	Holt 2020	2020	Men with normal or impaired Leydig cell function	Trial/report with sex steroid production outcomes	Vitamin D vs comparator	Candidate comparison retained for traceability	TT/sex steroid-related outcomes	Traceability record; not used in primary pooled outcome models	Included as traceability record to preserve 18-report/21-comparison mapping; final citation role should be checked before submission.

Supplementary Table S2. Included studies and comparison-level mapping — Outcome contribution

Comparison ID	Comparison label	TT primary	TT sensitivity/narrative	SHBG primary	SHBG sensitivity	FAI primary	FAI sensitivity	Calculated FT sensitivity	Direct FT narrative	BAT exploratory	Excluded from meta / notes
C01	Gheflati 2021	Yes		Yes		No					FAI scale incompatible
C02	Maghsoumi-Norouzabad 2021	Yes		Yes		Yes					
C03	Zittermann 2019	Yes		Yes	Yes			Yes		Yes	High RoB for TT
C04	Pilz 2011	Yes						Yes		Yes	High RoB; cointervention
C05	Saha 2018	Yes		Yes		Yes					Collapsed factorial contrast
C06	Ulrich 2021	Yes									Ulrich healthy cohort
C07	Ulrich 2021	Yes									Ulrich hemodialysis cohort
C08	Rips 2022	Yes									Active sport sensitivity flag
C09	Mielgo-Ayuso 2018	Yes									Elite athlete sensitivity flag
C10	Michalczyk 2020	Yes									Sport/cointervention sensitivity flag
C11	Ramezani Ahmadi 2020	Yes									Active male cohort
C12	Amini 2020	No		Yes		Yes			Yes		TT unit caution; direct FT not pooled
C13	Lerchbaum 2017	No			Yes	No		No			FAI scale and FT IQR concern
C14	Lerchbaum 2019	No			Yes		Yes	Yes			Median/IQR conversion
C15	Heijboer 2015	No	Yes								Heijboer source trial 1; not final-value primary
C16	Heijboer 2015	No	Yes								Heijboer source trial 2; not final-value primary
C17	Heijboer 2015	No	Yes								Heijboer source trial 3; not final-value primary

Comparison ID	Comparison label	TT primary	TT sensitivity/narrative	SHBG primary	SHBG sensitivity	FAI primary	FAI sensitivity	Calculated FT sensitivity	Direct FT narrative	BAT exploratory	Excluded from meta / notes
C18	Jorde 2013	No	Yes								Jorde change-score sensitivity
C19	Holt 2024	No	Yes								Holt 2024 CI inconsistency
C20	Jain 2024	No							Narrative		Jain ratio graphical / co-supplementation
C21	Holt 2020	No	Traceability only								Traceability record; not used in primary pooled outcome models

Supplementary Table S2. Included studies and comparison-level mapping — Coding legend

Field / term	Definition
Report	A publication or registry/report-level record retained after full-text screening.
Comparison-level record	An analytic unit created from a report when a male-specific population, source trial, factorial contrast, or clinically distinct cohort required separate handling.
Primary	Included in the main final-value model for the outcome.
Sensitivity	Included only in a restricted or secondary model because of conversion, method compatibility, or clinical/analytical caution.
Narrative	Retained for structured qualitative interpretation but not pooled.
Not pooled	Outcome data were absent, analytically incompatible, or affected by unresolved extractability concerns.
Collapsed factorial contrast	Multiple arms were combined using standard arm-combination rules to estimate one vitamin D versus no vitamin D contrast and avoid double counting.
Split comparison	A single report contributed more than one analytic comparison because clinically distinct male-specific cohorts were reported separately.

Supplementary Table S3. Extraction audit for TT, SHBG, FAI, FT, and BAT

Supplementary Table S3. Extraction audit for TT, SHBG, FAI, FT, and BAT — README

Supplementary Table S3 — Extraction audit	
Vitamin D Supplementation, Total Testosterone, and Androgen Bioavailability Markers in Adult Men: A Systematic Review and Meta-Analysis of Randomized Controlled Trials	
Item	Description
Purpose	Supplementary Table S3 provides an outcome-level extraction audit for TT, SHBG, FAI, free testosterone, and bioactive testosterone used or considered in the quantitative syntheses.
Effect direction	All mean differences are coded as vitamin D group minus control group. Positive values indicate higher final concentration/index values in the vitamin D group.
Primary TT model	Final total testosterone values were harmonised to nmol/L. Studies with ambiguous units or internally inconsistent dispersion were excluded from the primary TT model.
SHBG model	Primary conservative SHBG model used directly reported means/SDs or straightforward SE/CI-derived SDs. Median/IQR-derived studies were reserved for sensitivity analysis.
FAI model	Primary conservative FAI model retained only scale-compatible final-value data. Studies with probable simple-ratio or inconsistent scaling were excluded from primary pooling.
FT model	Directly measured FT and Vermeulen-calculated FT were not pooled together. The quantitative FT model is a method-restricted sensitivity analysis of calculated FT only.
BAT model	BAT was treated as exploratory because only two studies provided compatible extractable calculated BAT data.
Generated	2026-05-16 01:44

Supplementary Table S3. Extraction audit for TT, SHBG, FAI, FT, and BAT — TT extraction

Total testosterone extraction audit

Final-value TT data used in the primary model plus key studies excluded for extractability reasons.

Study/comparison	Population category	n VitD	Mean VitD	SD VitD	n Control	Mean Control	SD Control	Unit	Extraction/conversion	Analysis decision	RoB 2	Notes
Gheflati 2021	infertility	20	4.61	2.06	20	3.4	1.3	nmol/L	Direct/author-reported final value	Primary TT	Some concerns	Values from finalized manuscript primary TT extraction table
Maghsoumi-Norouzabad 2021	infertility	43	13.69	6.07	43	13.38	6.97	nmol/L	Direct/author-reported final value	Primary TT	Low risk	Values from finalized manuscript primary TT

Study/comparison	Population category	n VitD	Mean VitD	SD VitD	n Control	Mean Control	SD Control	Unit	Extraction/conversion	Analysis decision	RoB 2	Notes
												extraction table
Zittermann 2019	advanced heart failure	71	10	5.28	62	11.1	6.89	nmol/L	Direct/author-reported final value	Primary TT; high RoB sensitivity exclusion	High risk	Values from finalized manuscript primary TT extraction table
Pilz 2011	overweight/weight loss	31	13.4	4.7	23	12.7	5.5	nmol/L	Direct/author-reported final value	Primary TT; high RoB sensitivity exclusion	High risk	Values from finalized manuscript primary TT extraction table
Saha 2018	healthy young men	96	19.81	6.62	84	18.55	5.02	nmol/L	Collapsed factorial contrast	Primary TT	Some concerns	Collapsed cholecalciferol vs non-cholecalciferol contrast
Ulrich 2021a	healthy men	18	20.5	7.9	17	21.8	16.5	nmol/L	Split population comparison	Primary TT	Some concerns	Healthy-subject cohort treated as clinically distinct comparison
Ulrich 2021b	hemodialysis	9	7.8	3.8	9	11.6	4	nmol/L	Split population comparison	Primary TT; clinically extreme sensitivity exclusion	Some concerns	Hemodialysis cohort treated as clinically distinct comparison
Rips 2022	military/physically active	27	21.3	5.9	26	20.3	4.1	nmol/L	Direct/author-reported final value	Primary TT; athlete/military sensitivity exclusion	Some concerns	Values from finalized manuscript primary TT extraction table
Mielgo-Ayuso 2018	elite athletes	18	16.4	4.44	18	15.15	3.33	nmol/L	Direct/author-reported final value	Primary TT; athlete/military sensitivity exclusion	Some concerns	Values from finalized manuscript primary TT extraction table
Michalczyk 2020	elite athletes	15	28.25	3.2	13	26.6	5.1	nmol/L	Direct/author-reported final value	Primary TT; athlete/cointervention sensitivity exclusion	Some concerns	Values from finalized manuscript primary TT extraction table

Study/comparison	Population category	n VitD	Mean VitD	SD VitD	n Control	Mean Control	SD Control	Unit	Extraction/conversion	Analysis decision	RoB 2	Notes
Ramezani Ahmadi 2020	physically active men	20	18.24	3.5	20	20.8	9.01	nmol/L	Direct/author-reported final value	Primary TT; athlete/military sensitivity exclusion	Some concerns	Values from finalized manuscript primary TT extraction table
Amini 2020	infertile men	30			32			unit requires verification	Unit ambiguity	Excluded from primary TT; may be sensitivity only if unit verified	Not assessed for primary TT	Reported testosterone unit requires verification; not used in primary final-value model
Holt 2024	infertile men							nmol/L likely	Internally inconsistent CI/dispersion	Excluded from primary TT	Not assessed for primary TT	Placebo-arm final testosterone confidence interval internally inconsistent; author clarification required

Supplementary Table S3. Extraction audit for TT, SHBG, FAI, FT, and BAT — SHBG extraction

SHBG extraction audit

Final-value SHBG data with primary conservative versus sensitivity decisions.

Study/comparison	n VitD	Mean VitD	SD VitD	n Control	Mean Control	SD Control	Unit	Extraction status	Analysis decision	Notes
Gheflati 2021	20	47.82	30.63	20	32.73	15.92	nmol/L probable	SE to SD	Primary conservative	Unit-label flag: table label physiologically implausible but values consistent with nmol/L
Maghsoumi-Norouzabad 2021	43	19.09	3.69	43	18.88	4.71	nmol/L	Direct mean ± SD	Primary conservative	Direct extraction
Saha 2018	96	21.11	8.24	84	21.22	8.97	nmol/L	Collapsed factorial contrast	Primary conservative	Collapsed cholecalciferol vs non-cholecalciferol contrast
Amini 2020	30	25.76	9.99	32	26.68	13.53	nmol/L	Direct mean ± SD	Primary conservative	Study-level unit caution for TT does not require recalculating SHBG
Zittermann 2019	71	49	56.12	62	38.4	26.52	nmol/L	95% CI to SD	Primary conservative	Wide CI/dispersion caution

Study/comparison	n VitD	Mean VitD	SD VitD	n Control	Mean Control	SD Control	Unit	Extraction status	Analysis decision	Notes
Lerchbaum 2019	46	28.53	14	47	35.33	15.26	nmol/L	Median/IQR to mean/SD	Sensitivity only	Converted values used only in all-studies sensitivity analysis
Lerchbaum 2017	48	38.7	11.85	49	39.4	13.7	nmol/L	Median/IQR to mean/SD	Sensitivity only	Converted values used only in all-studies sensitivity analysis

Supplementary Table S3. Extraction audit for TT, SHBG, FAI, FT, and BAT — FAI extraction

Free Androgen Index extraction audit

Final-value FAI data and scale-compatibility decisions. Converted values are retained only as specified.

Study/comparison	n VitD	Mean VitD	SD VitD	n Control	Mean Control	SD Control	Unit/scale	Scale status	Conversion method	Analysis decision	Source location	Notes
Gheflati 2021	20	0.16	0.13416407865	20	0.26	0.4472135955	FAI reported as ratio of total testosterone to SHBG; no ×100 specified	probable_simple_ratio	SE_to_SD using SD = SE × sqrt(n)	Sensitivity only unless scale can be defended	Table 4, page 7: Free androgen index, After values	Do not include in primary FAI model. Final-value extraction uses After values. Reported as mean ± SE; SD converted.
Maghsoumi-Norouzabad 2021	43	40.56	22.71	43	39.13	23.72	FAI = T/SHBG ×100 (%)	compatible_x100	None; direct mean ± SD	Primary conservative	Table 2, page 7: FAI (T/SHBG.100) (%) 12 weeks	Eligible for primary conservative FAI model. Final-value extraction uses 12-week values.
Saha 2018	96	101.6083	35.3429	84	99.2202	35.5271	FAI, nmol/nmol; author-reported index calculated as total testosterone/SHBG	compatible_author_index_collapsed	Cochrane arm-combination formula: pooled n, weighted mean, and combined SD	Primary conservative after transparent factorial collapse	Table 2, page 7: Free androgen index final values at 6 months; Figure 1 gives final n per arm	VitD arms: cholecalciferol alone n=49, FAI 102.0±26.3; calcium+cholecalciferol n=47, FAI 101.2±43.1. No-VitD arms: double placebo n=43, FAI 102.1±33.3; calcium alone n=41, FAI 96.2±37.9.
Amini 2020	30	14.66	6.05	32	15.74	9.74	Author-reported FAI = total testosterone/SHBG ×100	reported_index_with_caution	None; direct extraction; no recalculation from TT	Primary with caution; sensitivity check recommended	Table 3, page 5: FAI after intervention values	Use only the author-reported FAI final values. Do not derive FAI from TT due to known TT-unit concerns.
Lerchbaum 2017	48	7.4	3.0393	49	6.8	3.4099	FAI reported by authors; formula states TT(nmol/L)/SHBG(nmol/L) ×100, but reported values are inconsistent with expected ×100 scale	uncertain_scale_converted_median_IQR	Mean≈median; SD≈(Q3-Q1)/1.349	Exclude from primary; do not pool with compatible ×100 FAI	Table 2, page 6: FAI study-end values; Methods page 3 defines FAI	Final values: vitamin D median 7.4 (IQR 5.5–9.6), placebo median 6.8 (IQR 5.0–9.6). Converted only for audit/sensitivity

Study/comparison	n VitD	Mean VitD	SD VitD	n Control	Mean Control	SD Control	Unit/scale	Scale status	Conversion method	Analysis decision	Source location	Notes
												exploration; not primary.
Lerchbaum 2019	46	46.7	15.8636	47	38.4	18.5322	FAI = TT/SHBG ×100; author-reported Free Androgen Index	compatible_x100_converted_median_IQR	Mean≈median; SD≈(Q3-Q1)/1.349	Sensitivity only; compatible scale but converted distribution	Table 2, page 6: Free Androgen Index study-end values; Procedures page 3 defines FAI	Final values: vitamin D median 46.7 (IQR 35.0–56.4), placebo median 38.4 (IQR 28.1–53.1). Sensitivity candidate because values are converted from median/IQR.

Supplementary Table S3. Extraction audit for TT, SHBG, FAI, FT, and BAT — FT extraction

Free testosterone extraction audit

FT classified by ascertainment method. Direct FT is not pooled with calculated FT.

Study/comparison	Population/context	Outcome	n VitD	VitD original	n Control	Control original	Original unit	Method/classification	Harmonized unit	Mean VitD	SD VitD	Mean Control	SD Control	Extractability	Analysis decision	Notes	Source location
Amini 2020	Infertile men; vitamin D3 vs placebo	Free testosterone	30	10.36 ± 3.41	32	11.91 ± 3.90	pg/mL	Directly measured FT; ECLIA	nmol/L	0.03591812	0.01182247	0.041291970000000004	0.0135213	Extractable	Do not combine with calculated FT	Unit conversion is straightforward, but direct ECLIA FT is not analytically equivalent to Vermeulen-calculated FT.	Amini 2020, Table 3/methods
Lerchbaum 2017	Men with low 25OHD; vitamin D vs placebo	Free testosterone	48	0.102 (IQR 0.009–0.131)	49	0.095 (IQR 0.075–0.119)	ng/mL	Calculated FT; Vermeulen	nmol/L	0.353634		0.329365	0.11308228317272052	Partially extractable	Exclude from quantitative FT model	VitD IQR lower bound appears internally questionable relative to median; SD conversion not reliable.	Lerchbaum 2017, Table 2
Lerchbaum 2019	Men; vitamin D vs placebo	Free testosterone	46	0.083 (IQR 0.059–0.096)	46	0.081 (IQR 0.057–0.093)	ng/mL	Calculated FT; Vermeulen	nmol/L	0.28776100000000004	0.09509191994069682	0.280827	0.09252186805040771	Extractable after median/IQR conversion	Eligible for sensitivity, not primary	Converted using mean≈median and SD≈IQR/1.349; compatible in method but converted data.	Lerchbaum 2019, Table 2
Gheflati 2021	Asthenozoospermia; vitamin D vs placebo	Free testosterone	20		20			Not reported						Not extractable	No FT data	Reports TT, SHBG and FAI only; no directly	Gheflati 2021,

Study/comparison	Population/context	Outcome	n VitD	VitD original	n Control	Control original	Original unit	Method/classification	Harmonized unit	Mean VitD	SD VitD	Mean Control	SD Control	Extractability	Analysis decision	Notes	Source location
																measured or calculated FT.	methods and Table 4
Maghsoumi-Norouzabad 2021	Asthenozoospermia; VD3 vs placebo	Free testosterone	43		43			Not reported						Not extractable	No FT data	Reports TT, SHBG, E2, PRO, LH, FSH, FAI, T/LH and T/E2; no FT.	Maghsoumi-Norouzabad 2021, Table 2
Zittermann 2019 / EVITA	Advanced heart failure; vitamin D3 4000 IU/day vs placebo for 3 years	Free testosterone	71	0.186 (95% CI 0.165–0.207)	62	0.211 (95% CI 0.180–0.243)	nmol/L	Calculated FT; Vermeulen	nmol/L	0.186	0.09028017614117523	0.211	0.12654655511804697	Extractable after CI-to-SD conversion	Eligible for sensitivity, not primary	Final follow-up mean and 95% CI converted to SD using SE≈CI width/3.92 and SD=SE×sqrt(n).	Zittermann 2019, Table 2
Pilz 2011	Overweight men in weight-reduction program; vitamin D3 vs placebo for 1 year	Free testosterone	31	0.267 ± 0.087	23	0.278 ± 0.097	nmol/L	Calculated FT; Vermeulen	nmol/L	0.267	0.087	0.278	0.097	Extractable	Eligible for sensitivity, not primary	Male subgroup; original trial was not sex-stratified at randomization and participants were in a weight-reduction program.	Pilz 2011, Table 1
Heijboer 2015	Post hoc analysis of three male intervention studies	Free testosterone						Not studied						Not extractable	No FT data	Authors explicitly state free testosterone concentrations were not studied.	Heijboer 2015, Discussion/limitations
Saha 2018	Young vitamin D-deficient men; 2×2 factorial cholecalciferol/calcium	Free testosterone						Not reported						Not extractable	No FT data	Reports total testosterone, SHBG and FAI; no FT or BAT.	Saha 2018, methods, Tables 1–2
Jain 2024	Vitamin D-deficient African Americans; placebo, LC, VD, VD+LC	Free/total testosterone ratio	23	Graphical only	22	Graphical only	ratio/index	Free/total testosterone ratio; mixed-sex; graphical						Not extractable for FT final-value	Exclude from FT meta-analysis	Reports free/total testosterone ratio and SHBG mainly graphically; VD+LC is a co-supplementation arm and not pure VD effect.	Jain 2024, Figure 3 and text

Supplementary Table S3. Extraction audit for TT, SHBG, FAI, FT, and BAT — BAT extraction

Bioactive testosterone extraction audit

BAT was considered exploratory because compatible extractable data were limited to two calculated BAT studies.

Study/comparison	Population/context	Outcome	n VitD	VitD original	n Control	Control original	Original unit	Method/classification	Harmonized unit	Mean VitD	SD VitD	Mean Control	SD Control	Extractability	Analysis decision	Notes	Source location
Pilz 2011	Overweight men in weight-reduction program; vitamin D3 vs placebo for 1 year	Bioactive testosterone	31	6.25 ± 2.01	23	6.59 ± 2.33	nmol/L	Calculated BAT; Vermeulen	nmol/L	6.25	2.01	6.59	2.33	Extractable	Eligible for exploratory BAT model	Male subgroup; weight-reduction program and no sex-stratified randomization.	Pilz 2011, Table 1
Zittermann 2019 / EVITA	Advanced heart failure; vitamin D3 4000 IU/day vs placebo for 3 years	Bioactive testosterone	71	4.39 (95% CI 3.82–4.95)	62	4.94 (95% CI 4.17–5.70)	nmol/L	Calculated BAT; Vermeulen	nmol/L	4.39	2.428966643798288	4.94	3.073273481438284	Extractable after CI-to-SD conversion	Eligible for exploratory BAT model	Final follow-up mean and 95% CI converted to SD using SE≈CI width/3.92 and SD=SE×sqrt(n).	Zittermann 2019, Table 2
Jain 2024	Vitamin D-deficient African Americans; placebo, LC, VD, VD+LC	Bioavailable testosterone						Not reported						Not extractable	No BAT data	Reports bioavailable 25(OH)D and free/total testosterone ratio, not bioavailable testosterone.	Jain 2024, Tables/Figures
Heijboer 2015	Post hoc analysis of three male intervention studies	Bioavailable/bioactive testosterone						Not reported						Not extractable	No BAT data	Focused on total testosterone; did not study free testosterone and no BAT extractable.	Heijboer 2015
Saha 2018	Young vitamin D-deficient men; 2×2 factorial cholecalciferol/calcium	Bioavailable/bioactive testosterone						Not reported						Not extractable	No BAT data	Reports total testosterone, SHBG and FAI only.	Saha 2018

Supplementary Table S3. Extraction audit for TT, SHBG, FAI, FT, and BAT — Meta analysis inputs

Meta-analysis input audit

R-ready input rows by outcome. yi and vi are formula-derived as VitD minus Control and variance of the mean difference.

Outcome	Study/comparison	Model role	n VitD	Mean VitD	SD VitD	n Control	Mean Control	SD Control	yi = Mean difference	vi = variance	Notes
Total testosterone	Gheflati 2021	primary	20	4.61	2.06	20	3.4	1.3	1.2100000000000004	0.29668	Primary TT
Total testosterone	Maghsoumi-Norouzabad 2021	primary	43	13.69	6.07	43	13.38	6.97	0.3099999999999987	1.986646511627907	Primary TT
Total testosterone	Zittermann 2019	primary	71	10	5.28	62	11.1	6.89	-1.0999999999999996	1.158332553384825	Primary TT; high RoB sensitivity exclusion
Total testosterone	Pilz 2011	primary	31	13.4	4.7	23	12.7	5.5	0.7000000000000011	2.0277980364656383	Primary TT; high RoB sensitivity exclusion
Total testosterone	Saha 2018	primary	96	19.81	6.62	84	18.55	5.02	1.259999999999998	0.7565089285714286	Primary TT
Total testosterone	Ulrich 2021a	primary	18	20.5	7.9	17	21.8	16.5	-1.3000000000000007	19.481928104575164	Primary TT
Total testosterone	Ulrich 2021b	primary	9	7.8	3.8	9	11.6	4	-3.8	3.382222222222222	Primary TT; clinically extreme sensitivity exclusion
Total testosterone	Rips 2022	primary	27	21.3	5.9	26	20.3	4.1	1	1.9357977207977208	Primary TT; athlete/military sensitivity exclusion
Total testosterone	Mielgo-Ayuso 2018	primary	18	16.4	4.44	18	15.15	3.33	1.2499999999999982	1.7112500000000002	Primary TT; athlete/military sensitivity exclusion
Total testosterone	Michalczyk 2020	primary	15	28.25	3.2	13	26.6	5.1	1.6499999999999986	2.6834358974358974	Primary TT; athlete/cointervention sensitivity exclusion
Total testosterone	Ramezani Ahmadi 2020	primary	20	18.24	3.5	20	20.8	9.01	-2.5600000000000023	4.671505	Primary TT; athlete/military sensitivity exclusion
SHBG	Gheflati 2021	primary	20	47.82	30.63	20	32.73	15.92	15.090000000000003	59.582164999999996	Primary conservative
SHBG	Maghsoumi-Norouzabad 2021	primary	43	19.09	3.69	43	18.88	4.71	0.21000000000000085	0.8325627906976745	Primary conservative
SHBG	Saha 2018	primary	96	21.11	8.24	84	21.22	8.97	-0.10999999999999943	1.6651345238095239	Primary conservative
SHBG	Amini 2020	primary	30	25.76	9.99	32	26.68	13.53	-0.9199999999999982	9.047323124999998	Primary conservative
SHBG	Zittermann 2019	primary	71	49	56.12	62	38.4	26.52	10.600000000000001	55.702228805088595	Primary conservative
SHBG	Lerchbaum 2019	sensitivity	46	28.53	14	47	35.33	15.26	-6.799999999999997	9.215499352451435	Sensitivity only
SHBG	Lerchbaum 2017	sensitivity	48	38.7	11.85	49	39.4	13.7	-0.6999999999999957	6.755876913265306	Sensitivity only

Outcome	Study/comparison	Model role	n VitD	Mean VitD	SD VitD	n Control	Mean Control	SD Control	yi = Mean difference	vi = variance	Notes
FAI	Gheflati 2021	sensitivity_candidate	20	0.16	0.13416407865	20	0.26	0.4472135955	-0.1	0.010900000000000205	Sensitivity only unless scale can be defended
FAI	Maghsoumi-Norouzabad 2021	primary_conservative	43	40.56	22.71	43	39.13	23.72	1.4299999999999997	25.07866279069767	Primary conservative
FAI	Saha 2018	primary_conservative	96	101.6083	35.3429	84	99.2202	35.5271	2.3880999999999943	28.0375635984375	Primary conservative after transparent factorial collapse
FAI	Amini 2020	primary_with_caution	30	14.66	6.05	32	15.74	9.74	-1.08	4.1846958333333335	Primary with caution; sensitivity check recommended
FAI	Lerchbaum 2017	excluded_from_primary_scale_uncertain	48	7.4	3.0393	49	6.8	3.4099	0.6000000000000005	0.4297389219770408	Exclude from primary; do not pool with compatible ×100 FAI
FAI	Lerchbaum 2019	sensitivity_candidate	46	46.7	15.8636	47	38.4	18.5322	8.300000000000004	12.778020780647548	Sensitivity only; compatible scale but converted distribution
Calculated FT	Lerchbaum 2019	sensitivity_only	46	0.287761	0.095092	46	0.280827	0.092522	0.006933999999999996	0.0003826697597391304	Vermeulen-calculated FT
Calculated FT	Zittermann 2019 / EVITA	sensitivity_only	71	0.186	0.09028	62	0.211	0.126547	-0.024999999999999994	0.00037308810282576097	Vermeulen-calculated FT
Calculated FT	Pilz 2011	sensitivity_only	31	0.267	0.087	23	0.278	0.097	-0.01100000000000001	0.0006532482468443198	Vermeulen-calculated FT
BAT	Pilz 2011	exploratory_only	31	6.25	2.01	23	6.59	2.33	-0.33999999999999986	0.3663649368863955	Vermeulen-calculated BAT
BAT	Zittermann 2019 / EVITA	exploratory_only	71	4.39	2.428967	62	4.94	3.073273	-0.5500000000000007	0.2354357325781638	Vermeulen-calculated BAT

Supplementary Table S3. Extraction audit for TT, SHBG, FAI, FT, and BAT — Decision log

Extraction and synthesis decision log

Key methodological decisions used to protect comparability and avoid unit-of-analysis errors.

Issue	Decision	Rationale
FT direct vs calculated	Not pooled together	Direct assay-based FT and equation-derived FT are analytically non-equivalent.
FAI scale incompatibility	Primary model restricted to compatible scale	FAI values were only pooled when scale compatibility was defensible.
Median/IQR data	Sensitivity only unless otherwise specified	Converted mean/SD values introduce distributional assumptions.

Issue	Decision	Rationale
Holt 2024 TT	Excluded from primary TT	Internal inconsistency in placebo-arm final CI/dispersion precluded reliable SD derivation.
Amini 2020 TT	Excluded from primary TT	Total testosterone unit requires verification; author-reported FAI and SHBG were retained where appropriate.
BAT	Exploratory	Only two studies reported compatible calculated BAT data.

Supplementary Table S4. RoB 2 domain-level matrix with rationales

Supplementary Table S4. RoB 2 domain-level matrix with rationales — Summary

Supplementary Table S4. RoB 2 domain-level matrix with rationales	
Scope	Outcome-specific RoB 2 assessment for final total testosterone comparisons included in the primary model.
Tool	Revised Cochrane risk-of-bias tool for randomized trials (RoB 2).
Outcome assessed	Final total testosterone concentration.
Number of comparisons	11
Low risk overall	1
Some concerns overall	8
High risk overall	2
High-risk comparisons	Zittermann 2019/EVITA; Pilz 2011.
Main reasons for concerns	Secondary/exploratory biomarker status, incomplete/per-protocol outcome data, male subgroup analyses, and cointerventions/clinical contexts.
Note	Judgements are outcome-specific and should not be interpreted as global article-level risk of bias.

Supplementary Table S4. RoB 2 domain-level matrix with rationales — RoB2 matrix

Study / comparison	D1 Randomization	D2 Deviations	D3 Missing outcome data	D4 Outcome measurement	D5 Selection of reported result	Overall RoB 2
Gheflati 2021	Some concerns	Low risk	Some concerns	Low risk	Some concerns	Some concerns
Maghsoumi-Norouzabad 2021	Low risk	Low risk	Low risk	Low risk	Low risk / minor concerns	Low risk
Zittermann 2019	Low risk / minor concerns	Low risk	High risk	Some concerns	Low risk	High risk
Pilz 2011	Some concerns	Low risk / minor concerns	Some concerns	Low risk	High risk / serious concerns	High risk
Saha 2018	Low risk	Some concerns	Some concerns	Low risk	Some concerns	Some concerns
Ulrich 2021a	Some concerns	Low risk	Some concerns	Low risk	Some concerns	Some concerns
Ulrich 2021b	Low risk	Low risk	Some concerns	Low risk	Some concerns	Some concerns
Rips 2022	Low risk	Low risk	Some concerns	Low risk	Some concerns	Some concerns
Mielgo-Ayuso 2018	Some concerns	Some concerns	Low risk	Low risk	Some concerns	Some concerns

Study / comparison	D1 Randomization	D2 Deviations	D3 Missing outcome data	D4 Outcome measurement	D5 Selection of reported result	Overall RoB 2
Michalczyk 2020	Some concerns	Some concerns	Some concerns	Low risk	Some concerns	Some concerns
Ramezani Ahmadi 2020	Low risk	Some concerns	Some concerns	Low risk	Some concerns	Some concerns

Supplementary Table S4. RoB 2 domain-level matrix with rationales — Domain rationales

Study / comparison	D1 rationale	D2 rationale	D3 rationale	D4 rationale	D5 rationale	Overall judgement	Senior notes
Gheflati 2021	Some concerns because randomization/allocation reporting was not sufficiently detailed for a low-risk judgement in the outcome-specific testosterone analysis.	Low risk: no major deviations from intended vitamin D versus control intervention were identified as likely to bias the testosterone estimate.	Some concerns because endocrine outcome data were secondary and completeness of outcome data was not fully robustly documented.	Low risk: total testosterone is an objective biochemical outcome.	Some concerns because testosterone appears as a secondary or exploratory endocrine marker rather than a clearly prespecified primary endpoint.	Some concerns	Retained in primary TT synthesis; judgement applies to final total testosterone outcome.
Maghsoumi-Norouzabad 2021	Low risk: randomized triple-blind placebo-controlled design and baseline balance/supporting reporting were sufficient for a low-risk judgement.	Low risk: blinded placebo-controlled intervention with no major deviation concerns identified.	Low risk: outcome data were judged sufficiently complete for the extracted testosterone outcome.	Low risk: total testosterone is an objective biochemical laboratory measure.	Low risk / minor concerns: endocrine outcomes were reported with sufficient transparency; only minor caution because androgen outcomes are biochemical secondary markers.	Low risk	Only comparison judged low risk overall for final total testosterone.
Zittermann 2019	Low risk / minor concerns: EVITA was randomized; only minor residual concerns relate to outcome-specific male subgroup handling.	Low risk: intervention delivery was unlikely to introduce bias in the testosterone comparison.	High risk due to substantial missing outcome data in the secondary analysis of advanced heart failure patients.	Some concerns because the outcome was a secondary biochemical marker in a clinically complex population.	Low risk: no clear evidence of selective reporting for the extracted testosterone result.	High risk	High-risk judgement driven primarily by D3 missing outcome data.
Pilz 2011	Some concerns because randomization reporting and allocation details were not sufficient for a fully low-risk judgement.	Low risk / minor concerns: intervention comparison was controlled; minor caution due to cointervention/context of weight loss.	Some concerns because the trial had small sample size and incomplete/per-protocol outcome considerations could affect precision and bias.	Low risk: total testosterone is an objective biochemical outcome.	High risk / serious concerns because testosterone analysis was not clearly prespecified and was conducted in a male subgroup of a broader weight-loss trial.	High risk	High-risk judgement driven primarily by D5 selective reporting/non-prespecified male subgroup analysis.
Saha 2018	Low risk: randomized factorial design was adequately described for the study-level intervention.	Some concerns because factorial cointerventions and collapsed vitamin D main-effect contrast require assumptions for the meta-analysis.	Some concerns because the testosterone analysis used a constructed collapsed contrast and outcome completeness was not fully ideal.	Low risk: total testosterone is an objective biochemical outcome.	Some concerns because testosterone was a secondary endocrine marker and the collapsed contrast was operationalized for synthesis.	Some concerns	Saha 2018 was handled as a collapsed cholecalciferol versus non-cholecalciferol factorial contrast.

Study / comparison	D1 rationale	D2 rationale	D3 rationale	D4 rationale	D5 rationale	Overall judgement	Senior notes
Ulrich 2021a	Some concerns because randomization/allocation details were not fully sufficient for the healthy male comparison.	Low risk: no major deviations from intended vitamin D supplementation were identified.	Some concerns because the healthy-subject comparison was small and outcome completeness details were limited.	Low risk: total testosterone is an objective biochemical outcome.	Some concerns because testosterone was a secondary biochemical outcome and comparison-level reporting required careful extraction.	Some concerns	Healthy-subject comparison split from Ulrich 2021.
Ulrich 2021b	Low risk: randomization was adequate for the dialysis comparison as reported.	Low risk: no major deviations from intended supplementation were identified.	Some concerns due to very small hemodialysis subgroup and limited outcome data robustness.	Low risk: total testosterone is an objective biochemical outcome.	Some concerns because testosterone was a secondary outcome and the comparison was small.	Some concerns	Hemodialysis comparison split from Ulrich 2021; clinically extreme population considered in sensitivity analysis.
Rips 2022	Low risk: randomized controlled design was sufficiently reported.	Low risk: intervention delivery did not raise major deviation concerns.	Some concerns because outcome data were secondary and attrition/completeness issues could not be fully excluded.	Low risk: total testosterone is an objective biochemical outcome.	Some concerns because testosterone was not the central endpoint and the population was physically active/military-like.	Some concerns	Included in athlete/military/active sport sensitivity exclusion.
Mielgo-Ayuso 2018	Some concerns because randomization/allocation reporting in the athlete context was not sufficiently detailed.	Some concerns because sport training/recovery context represents cointervention/exposure that may influence endocrine markers.	Low risk: extracted testosterone outcome data were sufficiently available.	Low risk: total testosterone is an objective biochemical outcome.	Some concerns because testosterone was a secondary marker in an elite-athlete intervention context.	Some concerns	Included in athlete/military/active sport sensitivity exclusion.
Michalczyk 2020	Some concerns because randomization/allocation reporting and baseline handling were not sufficient for a low-risk judgement.	Some concerns because sunlight exposure and oral D3 supplementation context may introduce cointervention or performance-training influences.	Some concerns because outcome completeness was not fully robust for the testosterone endpoint.	Low risk: total testosterone is an objective biochemical outcome.	Some concerns because testosterone was a secondary outcome and selective reporting could not be fully excluded.	Some concerns	Included in athlete/military/active sport and cointervention sensitivity exclusions.
Ramezani Ahmadi 2020	Low risk: randomized double-blind placebo-controlled design was sufficiently reported.	Some concerns because active/exercise context may influence endocrine markers and adherence/intervention deviations could not be fully ruled out.	Some concerns because outcome completeness for testosterone was not fully robustly documented.	Low risk: total testosterone is an objective biochemical outcome.	Some concerns because testosterone was one of several anabolic hormone outcomes and not the sole primary endpoint.	Some concerns	Included in athlete/active sport sensitivity exclusion.

Supplementary Table S4. RoB 2 domain-level matrix with rationales — Applicability notes

Item	Explanation
Outcome specificity	RoB 2 judgements apply to the final total testosterone outcome used in the primary meta-analysis. They should not be assumed to apply identically to SHBG, FAI, calculated FT, or BAT.

Item	Explanation
Unit-of-analysis	Multi-population and factorial studies were handled as comparison-level records only when justified by male-specific, clinically distinct, or collapsed-contrast data.
High-risk retention	High-risk studies were retained in the primary TT synthesis to preserve prespecified inclusion, with sensitivity analyses excluding high-risk studies.
Extractability vs risk of bias	Data validity problems such as ambiguous units, internally inconsistent confidence intervals, or non-comparable outcome scales were treated as extractability issues rather than RoB 2 judgements.
Interpretation	The RoB 2 profile contributes to GRADE downgrading for risk of bias and supports cautious interpretation of null pooled estimates.

Supplementary Table S4. RoB 2 domain-level matrix with rationales — Coding legend

Code	Meaning
D1	Bias arising from the randomization process.
D2	Bias due to deviations from intended interventions.
D3	Bias due to missing outcome data.
D4	Bias in measurement of the outcome.
D5	Bias in selection of the reported result.
Low risk	Information available indicates that bias is unlikely to materially affect the outcome-specific estimate.
Some concerns	Some limitations or incomplete information exist, but they do not clearly justify a high-risk judgement.
High risk	A serious concern was identified that could plausibly bias the outcome-specific estimate.
Low risk / minor concerns	Used when the available information was close to low risk but minor reporting uncertainty remained.
High risk / serious concerns	Used when serious concerns were concentrated in a specific domain and determined the overall high-risk judgement.

Supplementary Table S5. GRADE downgrading details by domain

Supplementary Table S5. GRADE downgrading details by domain — Summary

Supplementary Table S5	GRADE downgrading details
Project	Vitamin D Supplementation, Total Testosterone, and Androgen Bioavailability Markers in Adult Men: A Systematic Review and Meta-Analysis of Randomized Controlled Trials
Purpose	To document outcome-specific GRADE certainty judgements and downgrading decisions.
Evidence base	Randomized controlled trials; certainty started at high and was downgraded by GRADE domains.
Outcomes assessed	Total testosterone; SHBG; Free Androgen Index; calculated free testosterone; bioactive testosterone
Primary interpretation	Current randomized evidence does not demonstrate a clear or reproducible effect of vitamin D supplementation on androgen bioavailability markers.
Important methodological constraint	Directly measured free testosterone and calculated free testosterone were not pooled because they are analytically non-equivalent.
Date generated	2026-05-16

Supplementary Table S5. GRADE downgrading details by domain — GRADE downgrading

Outcome	Studies	Participants	Model role	Effect estimate	Starting certainty	Risk of bias	Inconsistency	Indirectness	Imprecision	Publication bias	Final certainty	Explanation
Total testosterone	11	703	Secondary biochemical anchor; primary final-value TT model	MD 0.47 nmol/L (95% CI -0.50 to 1.44)	High	Serious	Not serious	Not serious	Not serious to serious	Not assessable / undetected	Low	Downgraded mainly for risk of bias and imprecision. Most comparisons had some concerns or high risk of bias, and the confidence interval crossed the null. Indirectness was not judged serious within the broad adult male framework prespecified for this review.
SHBG	5	501	Primary conservative	MD 0.27 nmol/L (95% CI -2.14 to 2.68)	High	Serious	Not serious	Not serious	Not serious	Not assessable / undetected	Low	Downgraded mainly for risk of bias and imprecision.

Outcome	Studies	Participants	Model role	Effect estimate	Starting certainty	Risk of bias	Inconsistency	Indirectness	Imprecision	Publication bias	Final certainty	Explanation
			bioavailability-marker analysis									SHBG was a prespecified androgen-bioavailability marker, the effect estimate was consistent with no observed statistical heterogeneity, and indirectness was not judged serious within the review framework.
Free Androgen Index	3	328	Primary conservative FAI model	MD -0.37 (95% CI -4.28 to 3.55)	High	Serious	Not serious in primary model	Not serious for the primary model based on comparable FAI scaling	Serious	Not assessable	Low	Downgraded for risk of bias and imprecision because only three comparisons contributed to the primary conservative model. Indirectness was not judged serious for the primary model because only scale-compatible FAI values were pooled; non-comparable scales were excluded.
Calculated free testosterone	3	279	Method-restricted sensitivity analysis; calculated FT only	MD -0.010 nmol/L (95% CI -0.053 to 0.033)	High	Serious	Not serious	Serious	Serious	Not assessable	Very low	Downgraded because the analysis was restricted to calculated FT studies and does not represent all FT evidence. Direct FT was not pooled with calculated FT. The evidence base was small and

Outcome	Studies	Participants	Model role	Effect estimate	Starting certainty	Risk of bias	Inconsistency	Indirectness	Imprecision	Publication bias	Final certainty	Explanation
												method-constrained.
Bioactive testosterone	2	187	Exploratory analysis	MD -0.47 nmol/L (95% CI -1.77 to 0.83)	High	Serious	Not serious, but underpowered	Serious	Very serious	Not assessable	Very low	Downgraded for risk of bias, indirectness, and very serious imprecision because only two studies provided compatible data. The analysis was explicitly exploratory and underpowered to evaluate inconsistency or publication bias.

Supplementary Table S5. GRADE downgrading details by domain — SoF table

Outcome	Studies / participants	Effect estimate	Certainty of evidence	Interpretation
Total testosterone	11 studies / 703 participants	MD 0.47 nmol/L<br>95% CI -0.50 to 1.44	Low	Vitamin D supplementation did not show a clear effect on final total testosterone concentrations.
SHBG	5 studies / 501 participants	MD 0.27 nmol/L<br>95% CI -2.14 to 2.68	Low	Vitamin D supplementation did not show a clear effect on final SHBG concentrations.
Free Androgen Index	3 studies / 328 participants	MD -0.37<br>95% CI -4.28 to 3.55	Low	Vitamin D supplementation did not show a clear effect on FAI in the conservative primary model.
Calculated free testosterone	3 studies / 279 participants	MD -0.010 nmol/L<br>95% CI -0.053 to 0.033	Very low	Sensitivity-only analysis restricted to calculated FT showed no clear effect.
Bioactive testosterone	2 studies / 187 participants	MD -0.47 nmol/L<br>95% CI -1.77 to 0.83	Very low	Exploratory analysis showed no clear effect, but certainty is very limited.

Supplementary Table S5. GRADE downgrading details by domain — Coding legend

Term / judgement	Definition / application in this review
High certainty	Randomized evidence starts at high certainty before downgrading.

Term / judgement	Definition / application in this review
Moderate certainty	Not assigned in the current evidence profile.
Low certainty	The true effect may be substantially different from the pooled estimate; used for total testosterone, SHBG, and FAI.
Very low certainty	The true effect is likely to be substantially different from the estimate; used for calculated FT and BAT.
Risk of bias	Downgraded when most comparisons had some concerns or high risk of bias, or when outcome data were secondary/subgroup-based.
Inconsistency	Assessed using consistency of point estimates, heterogeneity statistics, and model interpretation; not downgraded for outcomes with no observed heterogeneity, but small k was considered in imprecision.
Indirectness	Assessed at the outcome level. Not judged serious for TT, SHBG, or the primary scale-compatible FAI model; judged serious for method-restricted calculated FT and exploratory BAT analyses.
Imprecision	Downgraded when few studies/participants contributed data, confidence intervals crossed the null, or the evidence base was exploratory.
Publication bias	Generally not formally assessable due to small numbers of studies by outcome.
Calculated FT	Assessed only as a method-restricted sensitivity analysis. Directly measured FT was not pooled with calculated FT.
BAT	Bioactive testosterone; handled as exploratory because only two compatible studies contributed data.

Supplementary Table S5. GRADE downgrading details by domain — Methodological notes

Item	Methodological note
Scope	This table documents GRADE judgements for the outcomes reported in the manuscript: total testosterone, SHBG, FAI, calculated FT, and BAT.
Outcome hierarchy	SHBG and FAI are the primary androgen-bioavailability markers; total testosterone is a secondary biochemical anchor; calculated FT is sensitivity-only; BAT is exploratory.
FT handling	Directly measured FT and calculated FT were not pooled together because they are non-equivalent ascertainment methods.
FAI handling	FAI certainty accounts for scale compatibility issues. The primary conservative model excluded non-comparable FAI scales.
BAT handling	BAT certainty is very low because only two compatible studies were available, making the analysis exploratory.
Publication bias	Formal publication-bias assessment was not considered reliable for outcomes with small numbers of contributing studies.
Manuscript consistency	The final certainty ratings match the Summary of Findings table and manuscript text: low for total testosterone, SHBG, and FAI; very low for calculated FT and BAT.

Supplementary Figures

Supplementary Figure S1. Calculated free testosterone sensitivity analysis.

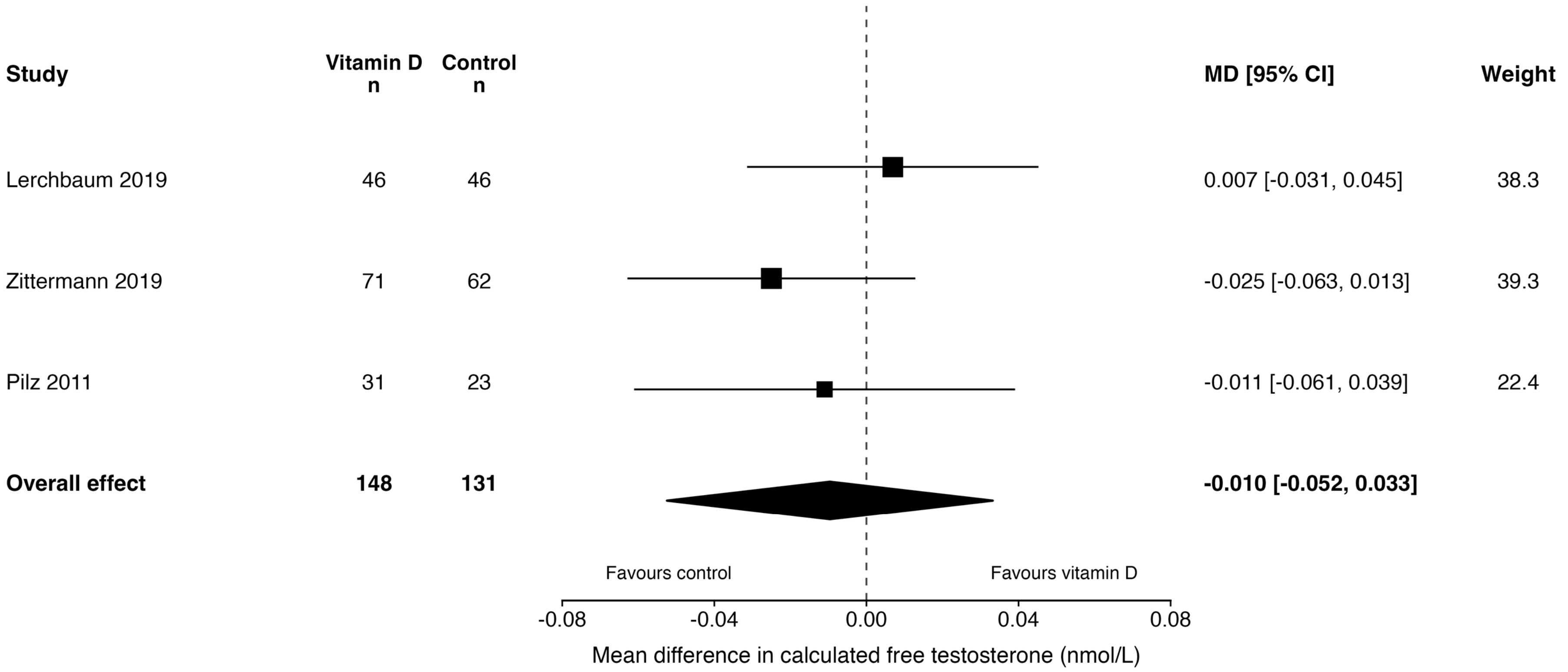

Supplementary Figure S1. Calculated free testosterone sensitivity analysis.

Supplementary Figure S2. Bioactive testosterone exploratory analysis.

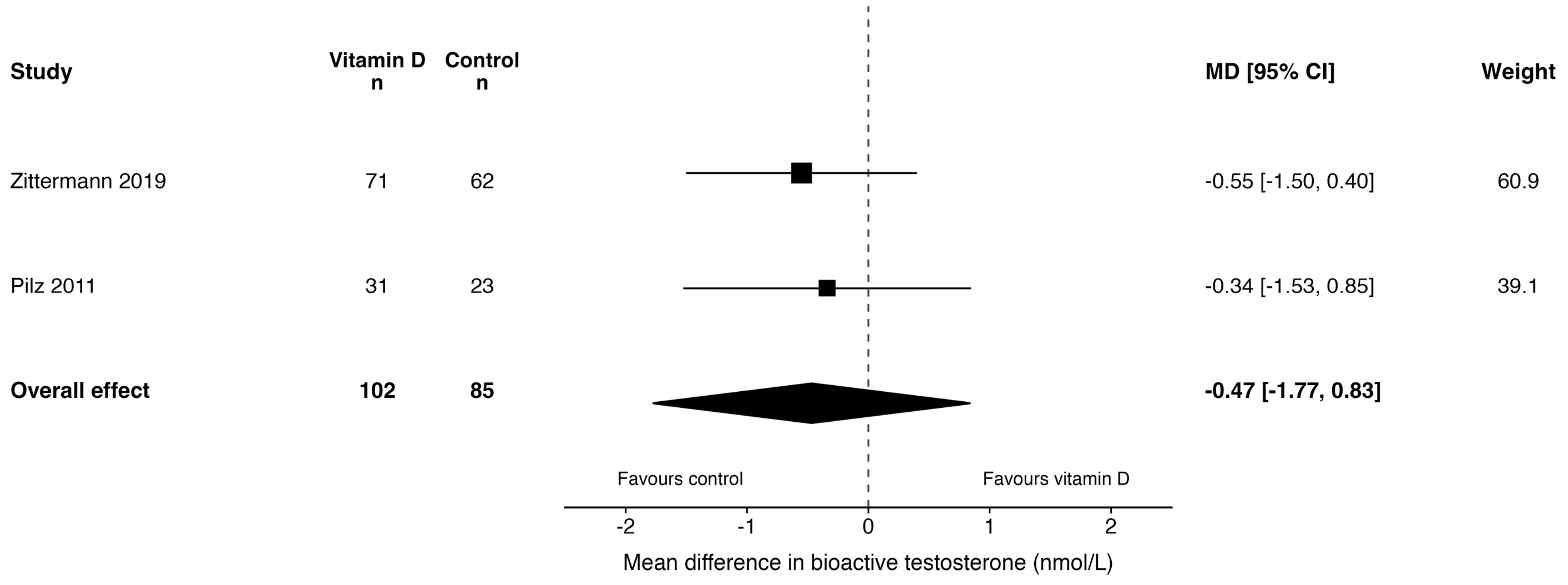

Supplementary Figure S2. Bioactive testosterone exploratory analysis.

Supplementary Figure S3. RoB 2 outcome-level matrix for total testosterone.

	D1	D2	D3	D4	D5	Overall
Gheflati 2021	Some concerns	Low	Some concerns	Low	Some concerns	**Some concerns**
Maghsoumi 2021	Low	Low	Low	Low	Low	**Low**
Zittermann 2019	Low	Low	**High**	Some concerns	Low	**High**
Pilz 2011	Some concerns	Low	Some concerns	Low	**High**	**High**
Saha 2018	Low	Some concerns	Some concerns	Low	Some concerns	**Some concerns**
Ulrich 2021a	Some concerns	Low	Some concerns	Low	Some concerns	**Some concerns**
Ulrich 2021b	Low	Low	Some concerns	Low	Some concerns	**Some concerns**
Rips 2022	Low	Low	Some concerns	Low	Some concerns	**Some concerns**
Mielgo 2018	Some concerns	Some concerns	Low	Low	Some concerns	**Some concerns**
Michalczyk 2020	Some concerns	Some concerns	Some concerns	Low	Some concerns	**Some concerns**
Ramezani 2020	Low	Some concerns	Some concerns	Low	Some concerns	**Some concerns**

D1: randomization process; D2: deviations from intended interventions; D3: missing outcome data; D4: outcome measurement; D5: selection of the reported

Supplementary Figure S3. RoB 2 outcome-level matrix for total testosterone.

Supplementary Figure S4, bioactive testosterone exploratory forest plot.

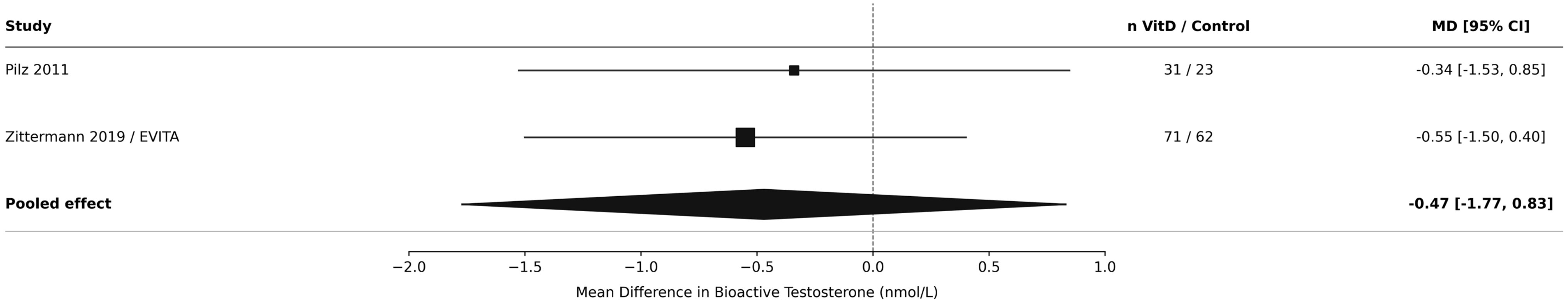

Supplementary Figure S5, Summary of findings and GRADE certainty of evidence figure.

Summary of Findings and GRADE Certainty of Evidence

Vitamin D supplementation and androgen bioavailability markers in adult men

Outcome	Studies	Participants	Effect estimate	Certainty	Interpretation
Total testosterone	11	703	MD 0.47 nmol/L (95% CI -0.50 to 1.44)	**LOW**	No clear effect on final total testosterone concentrations.
SHBG	5	501	MD 0.27 nmol/L (95% CI -2.14 to 2.68)	**LOW**	No clear effect on final SHBG concentrations.
Free Androgen Index	3	328	MD -0.37 (95% CI -4.28 to 3.55)	**LOW**	No clear effect on FAI in the conservative primary model.
Calculated free testosterone	3	279	MD -0.010 nmol/L (95% CI -0.053 to 0.033)	**VERY LOW**	Sensitivity-only analysis restricted to calculated FT showed no clear effect.
Bioactive testosterone	2	187	MD -0.47 nmol/L (95% CI -1.77 to 0.83)	**VERY LOW**	Exploratory analysis showed no clear effect, but certainty is very limited.

Abbreviations: CI, confidence interval; FAI, Free Androgen Index; FT, free testosterone; MD, mean difference; SHBG, sex hormone-binding globulin.

GRADE notes: Downgrading decisions were based on risk of bias, indirectness, imprecision, and, where applicable, limited compatibility of ascertainment methods or small-study evidence.

Calculated free testosterone was assessed only as a method-restricted sensitivity analysis. Bioactive testosterone was considered exploratory because only two studies provided compatible extractable data.

PRISMA 2020 Checklist

PRISMA item	Topic	Reported location	Notes
1	Title	Title page	Complete; update page number after final pagination.
2	Abstract	Structured Abstract	Complete; update page number after final pagination.
3	Rationale	Introduction	Complete; update page number after final pagination.
4	Objectives	End of Introduction	Complete; update page number after final pagination.
5	Eligibility criteria	Materials and Methods: Eligibility criteria	Complete; update page number after final pagination.
6	Information sources	Materials and Methods: Information sources and search strategy; Supplementary File S1	Complete; update page number after final pagination.
7	Search strategy	Supplementary File S1; Materials and Methods: Information sources and search strategy	Complete; update page number after final pagination.
8	Selection process	Materials and Methods: Selection process and PRISMA reconciliation	Complete; update page number after final pagination.
9	Data collection process	Materials and Methods: Data extraction and quantitative decision rules	Complete; update page number after final pagination.
10a	Data items – outcomes	Materials and Methods: Eligibility criteria; Data extraction and quantitative decision rules	Complete; update page number after final pagination.
10b	Data items – other variables	Materials and Methods: Data extraction and quantitative decision rules	Complete; update page number after final pagination.
11	Study risk of bias assessment	Materials and Methods: Risk-of-bias assessment; Supplementary Table S4; Figure S3	Complete; update page number after final pagination.
12	Effect measures	Materials and Methods: Effect measures and data synthesis	Complete; update page number after final pagination.
13a	Synthesis methods – eligibility for synthesis	Study-level and comparison-level handling; Data extraction and quantitative decision rules	Complete; update page number after final pagination.
13b	Synthesis methods – data preparation	Data extraction and quantitative decision rules; Supplementary Table S3	Complete; update page number after final pagination.
13c	Synthesis methods – tabulation/visual display	Results; Figures 2–4; Supplementary Figures S1–S3	Complete; update page number after final pagination.
13d	Synthesis methods – statistical methods	Effect measures and data synthesis	Complete; update page number after final pagination.
13e	Synthesis methods – heterogeneity	Effect measures and data synthesis; Results	Complete; update page number after final pagination.
13f	Synthesis methods – sensitivity analyses	Effect measures and data synthesis; Results	Complete; update page number after final pagination.
14	Reporting bias assessment	Effect measures and data synthesis; GRADE certainty of evidence and Summary of Findings	Complete; update page number after final pagination.
15	Certainty assessment	Materials and Methods: Certainty of evidence; Table 2; Supplementary Table S5	Complete; update page number after final pagination.

16a	Study selection – results	Results: Study selection and full-text audit; Figure 1	Complete; update page number after final pagination.
16b	Study selection – exclusions	Results: Study selection and full-text audit; Supplementary Table S1	Complete; update page number after final pagination.
17	Study characteristics	Results; Supplementary Table S2	Complete; update page number after final pagination.
18	Risk of bias in studies	Results: Risk-of-bias results; Supplementary Table S4; Figure S3	Complete; update page number after final pagination.
19	Results of individual studies	Results tables; Figures 2–4; Supplementary Figures S1–S2	Complete; update page number after final pagination.
20a	Results of syntheses – summary characteristics	Results: Quantitative synthesis sections	Complete; update page number after final pagination.
20b	Results of syntheses – statistical results	Results; Figures 2–4; Supplementary Figures S1–S2	Complete; update page number after final pagination.
20c	Results of syntheses – heterogeneity	Results: Quantitative synthesis sections	Complete; update page number after final pagination.
20d	Results of syntheses – sensitivity analyses	Results: Total testosterone, SHBG, FAI, calculated FT and BAT sections	Complete; update page number after final pagination.
21	Reporting biases	Small-study effects and publication bias statement; GRADE section	Complete; update page number after final pagination.
22	Certainty of evidence	Table 2; GRADE certainty of evidence and Summary of Findings	Complete; update page number after final pagination.
23a	Discussion – general interpretation	Discussion	Complete; update page number after final pagination.
23b	Discussion – limitations of evidence	Discussion; Limitations paragraph	Complete; update page number after final pagination.
23c	Discussion – limitations of review process	Discussion; Limitations paragraph	Complete; update page number after final pagination.
23d	Discussion – implications	Discussion; Conclusions	Complete; update page number after final pagination.
24a	Registration and protocol	Materials and Methods: Protocol and registration	Complete; update page number after final pagination.
24b	Protocol access	Materials and Methods: Protocol and registration	Complete; update page number after final pagination.
24c	Protocol amendments	Materials and Methods: Protocol and registration	Complete; update page number after final pagination.
25	Support	Funding	Complete; update page number after final pagination.
26	Competing interests	Conflicts of Interest	Complete; update page number after final pagination.
27	Availability of data, code and other materials	Data Availability Statement; Supplementary Materials	Complete; update page number after final pagination.
